# Targeting Cancer Resistance via Multifunctional Gold Nanoparticles

**DOI:** 10.3390/ijms20215510

**Published:** 2019-11-05

**Authors:** Pedro Pedrosa, M. Luísa Corvo, Margarida Ferreira-Silva, Pedro Martins, Manuela Colla Carvalheiro, Pedro M. Costa, Carla Martins, L. M. D. R. S. Martins, Pedro V. Baptista, Alexandra R. Fernandes

**Affiliations:** 1 Applied Molecular Biosciences Unit (UCIBIO), Departamento de Ciências da Vida, Faculdade de Ciências e Tecnologia, Universidade NOVA de Lisboa, Campus de Caparica, 2829-516 Caparica, Portugal; pm.pedrosa@campus.fct.unl.pt (P.P.); margarida_ferreira_21@hotmail.com (M.F.-S.); pvb0608@gmail.com (P.M.); pmcosta@fct.unl.pt (P.M.C.); c.martins@campus.fct.unl.pt (C.M.); 2 Instituto de Investigação do Medicamento (iMed.ULisboa), Faculdade de Farmácia, Universidade de Lisboa, Av. Prof. Gama Pinto, 1649-003 Lisboa, Portugal; lcorvo@ff.ulisboa.pt (M.L.C.); mcarvalheiro@ff.ulisboa.pt (M.C.C.); 3 Centro de Química Estrutural (CQE), Instituto Superior Técnico, Av. Rovisco Pais, 1049-001 Lisboa, Portugal; luisamargaridamartins@tecnico.ulisboa.pt

**Keywords:** gold nanoparticles, cancer, cancer resistance, nanomedicine

## Abstract

Resistance to chemotherapy is a major problem facing current cancer therapy, which is continuously aiming at the development of new compounds that are capable of tackling tumors that developed resistance toward common chemotherapeutic agents, such as doxorubicin (DOX). Alongside the development of new generations of compounds, nanotechnology-based delivery strategies can significantly improve the in vivo drug stability and target specificity for overcoming drug resistance. In this study, multifunctional gold nanoparticles (AuNP) have been used as a nanoplatform for the targeted delivery of an original anticancer agent, a Zn(II) coordination compound [Zn(DION)2]Cl2 (ZnD), toward better efficacy against DOX-resistant colorectal carcinoma cells (HCT116 DR). Selective delivery of the ZnD nanosystem to cancer cells was achieved by active targeting via cetuximab, NanoZnD, which significantly inhibited cell proliferation and triggered the death of resistant tumor cells, thus improving efficacy. In vivo studies in a colorectal DOX-resistant model corroborated the capability of NanoZnD for the selective targeting of cancer cells, leading to a reduction of tumor growth without systemic toxicity. This approach highlights the potential of gold nanoformulations for the targeting of drug-resistant cancer cells.

## 1. Introduction

Chemotherapy agents, such as doxorubicin (DOX), are frequently used in first-line therapy against colorectal and lung cancer, but the efficacy of these agents is limited by the development of drug resistance. This is a major hurdle to improving treatment outcomes and extending the overall survival of patients. One way to overturn this drawback has been via the synthesis of novel compounds and their incorporation in innovative formulations that are suitable for the selective delivery to cancer cells, thus optimizing the therapeutic window and circumventing drug resistance to conventional chemotherapeutics. Among these novel compounds, we have been focusing on the study of the antiproliferative potential and respective mechanisms of action of coordination compounds associated with suitable targeted delivery platforms [1,2,3,4,5]. One such compound, [Zn(DION)_2_]Cl_2_—ZnD (DION—1,10-phenanthroline-5,6-dione), showed a high cytotoxic potential toward cancer cell lines, with IC_50_ values 2x and 70x lower than those of doxorubicin (DOX) and cisplatin, respectively [1].

Recent advances in cancer nanomedicine have provided a wide range of tools suitable for the directed and selective delivery of drugs to cancer cells, such as multifunctional gold nanoparticles (AuNPs) [6,7,8]. The development of nanovectorization platforms for the targeted delivery of chemotherapeutics has been able to enhance therapeutic efficacy while circumventing drug resistance [5,7,8]. For example, the epidermal growth factor receptor (EGFR) is frequently overexpressed in cancer cells and may be used to selectively target nanosystems (or drugs) via the therapeutic monoclonal antibody cetuximab (the FDA-approved drug for the treatment of colorectal and lung cancer) [9,10,11,12,13,14,15,16,17,18,19,20,21,22]. Cetuximab blocks the receptor-dependent signal transduction of EGFR, leading to cell-cycle arrest, the induction of apoptosis, inhibition of angiogenesis, inhibition of metastasis, internalization, and self-downregulation [23]. In fact, EGFR-binding antibodies have been used as active targeting moieties in nanoparticle formulation, directing these nanovectors to interact with the cancer cells, thus reducing the deleterious impact to healthy cells and greatly improving the therapeutic index [24].

**Figure 1:**
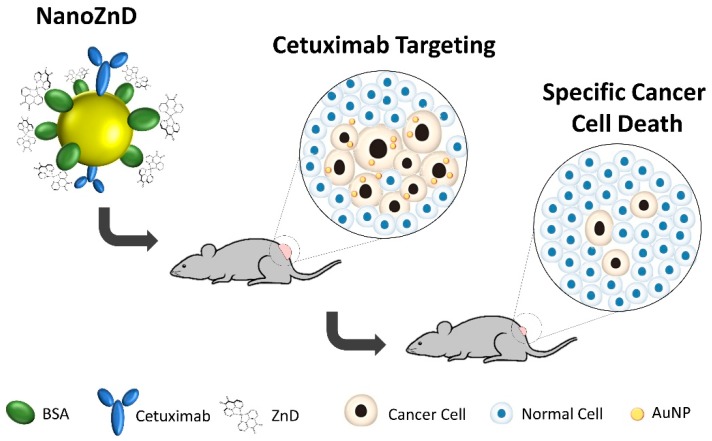
Schematic representation of the NanoZnD strategy. The gold nanoparticle (AuNP) core is covered by polyethylene glycol (PEG) for improved biocompatibility and stability in complex media; the antiproliferative compound—ZnD ([Zn(DION)_2_]Cl_2_)—is embedded within bovine serum albumin (BSA) and cetuximab for targeting.

Here, we present a novel nanovectorization system for the selective delivery of ZnD to DOX-resistant colorectal carcinoma cells, which is capable of tackling cancer development and increasing efficacy in vivo without impacting the healthy tissue (Figure 1).

## 2. Results and Discussion

### 2.1. ZnD as a Powerful Anticancer Compound

The synthesis and characterization of novel anticancer compounds usually requires a comprehensive understanding of the mechanism of cell death to fully take advantage of the compounds’ characteristics. ZnD was previously synthetized and characterized as a water-soluble compound [1]. We confirmed its stability by UV-Vis, for a 48-h period in water and phosphate-buffered saline (PBS) and in biological medium (Supplementary Figure S1). ZnD was also described as highly cytotoxic to HCT116 colorectal carcinoma cells with an IC_50_ of 0.217 ± 0.022 μM, compared to ZnCl_2_ (IC_50_ >100 μM) or the DION alone (IC_50_ >0.68 μM) [1]. The ZnD IC_50_ of human primary fibroblasts (IC_50_ 0.60 ± 0.13 μM) [1] demonstrates some selectivity toward cancer cells, i.e., requires lower amounts of chemical to kill cancer cells without adverse effects to healthy cells.

**Figure 2:**
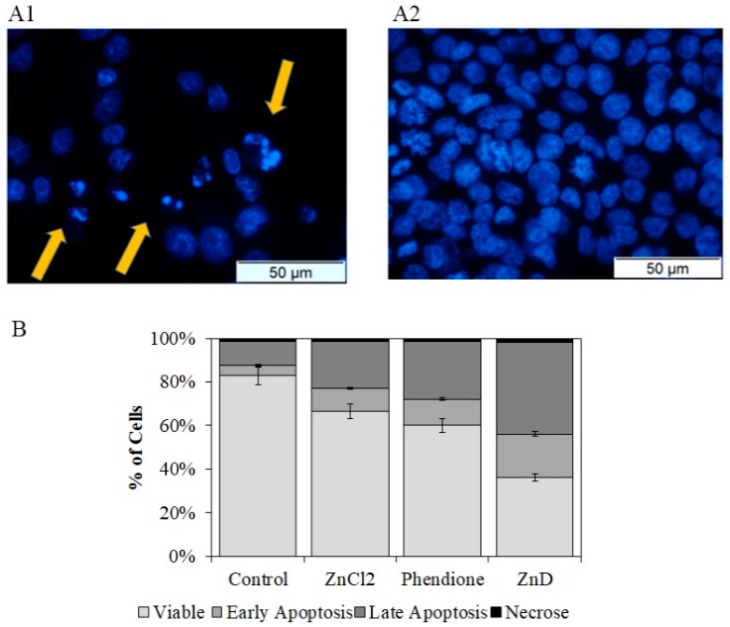

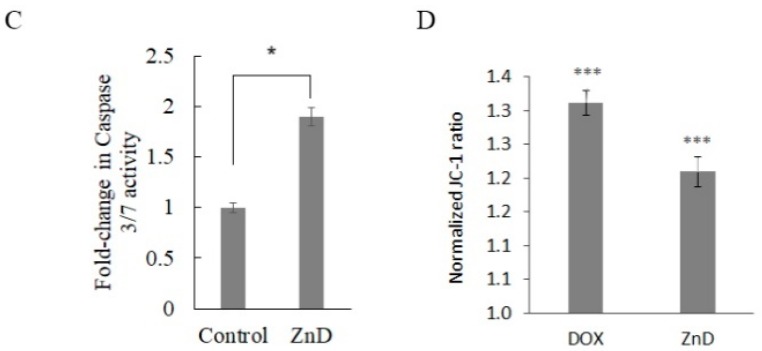
Evaluation of cell death mechanisms. (**A**) Morphological alterations observed after nuclei staining with DNA-specific dye Hoechst 33258 in HCT116 cells exposed to ZnD (IC_50_; 0.217 µM) (**A1**) for 48 h compared to vehicle control (sterile water, **A2**). Orange arrowheads indicate major alterations in nuclear morphology such as chromatin condensation and nuclear fragmentation. (**B**) Caspase 3/7 activity of HCT116 cell exposed to ZnD (IC_50_) or vehicle control (water). The results are expressed as the mean ± SEM percentage normalized to controls from three independent experiments. (**C**) Proportion of apoptotic and necrotic HCT116 cells after a period of incubation (48 h) in the presence of vehicle control (water), ZnCl_2_ (0.22 µM), DION (0.44 µM), and ZnD (IC_50_ = 0.217 µM). Cell death was determined by flow cytometry after Annexin-V/FITC (fluorescein isothiocyanate) and propidium iodide (PI) double staining. Light gray—viable cells; gray—early apoptosis; dark gray—late apoptosis; black—necrosis. (**D**) Mitochondrial membrane potential of HCT116 cells challenged with ZnD. The fluorescence of the 5,5,6,6-tetrachloro-1,1,3,3 tetraethylbenzimidazolylcarbocyanine iodide (JC-1) monomer, corresponding to green fluorescence, was acquired using filter BL1 (excitation and emission range wavelengths of 488 nm and 515–545 nm, respectively). The fluorescence of the JC-1 aggregate, corresponding to red fluorescence, was acquired using filter BL2 (excitation and emission range wavelengths of 488 nm and 561–587 nm, respectively) JC-1 monomer/aggregate ratio obtained after 48 h incubation of HCT116 cells with doxorubicin and ZnD. Values were normalized to the JC-1 ratio of the control and are represented as the mean ± SD of three independent experiments. * *p* < 0.05 compared to control; *** *p* < 0.0005 relative to control.

When addressing drug resistance, understanding the mechanism of action of candidate compounds allows identifying the pathways that can circumvent the known drug-resistant mechanisms triggered by the conventional therapy. For that purpose, cellular and molecular targets of ZnD were characterized. When cells are exposed to the IC_50_ of ZnD for 48 h, chromatin condensation and nuclear fragmentation was observed (Figure 2A), indicating possible genotoxic events. Additionally, by double-staining cells with propidium iodide (PI) and annexin V-FITC (fluorescein isothiocyanate) (Figure 2B), we observed that the number of apoptotic cells, either in early (FITC+/PI–) or later stages (FITC+/PI+), increased four-fold with only ~2% of cells in necrosis when compared to the control. This prompted us to evaluate the activation of the apoptotic pathway, which showed a two-fold increase of caspase-3/7 activity in HCT116 exposed to ZnD (Figure 2C). Together, these results confirm that ZnD induces HCT116 cell death via activation of the apoptotic pathway. The assessment of the mitochondrial membrane potential in these cells showed a depolarization of the membrane potential (in similar levels to those induced by DOX) that agrees with the induction of the intrinsic apoptotic pathway (Figure 2D and Figure S2).

**Figure 3:**
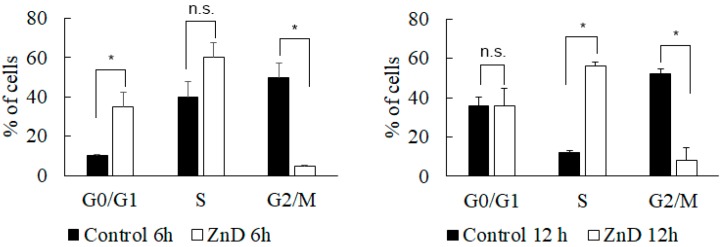
Cell cycle evaluation. Percentage of HCT116 cells in the different phases of the cell cycle after incubation for 6 and 12 h in the presence (0.217 µM, IC_50_ at 48 h) or absence (water) of the ZnD compound. PI fluorescent levels were determined by flow cytometry. The results are expressed as the mean ± SEM percentage from three independent experiments. * *p* < 0.05 compared to control. n.s.—no statistical difference.

Regarding the capability of ZnD to disrupt the cell cycle, data showed that ZnD induced cell cycle arrest, preventing cells from entering G2/mitosis (Figure 3). Indeed, most of the ZnD-treated HCT116 cells are in the S-phase, while untreated cells have freely progressed to G2/mitosis. These findings prompted us to evaluate the capability of ZnD to induce chromosomal alterations. Chinese hamster pulmonary fibroblasts (V79) treated with IC_50_ of ZnD for 24 h (using mitomycin C as positive control) showed no alterations in chromosome number or structure in the presence of ZnD (Supplementary Figure S3A). The absence of marked genotoxic effects was corroborated via the comet assay, where the DNA percentage in the tail was used as a measure for total DNA strand breakage (Figure S3B,C) and showed similar profiles for ZnD and for the control. Together, these results show the effective cytotoxic and cytostatic capability of ZnD but without significant genotoxicity in HCT116 cells.

### 2.2. ZnD and DOX-Resistant Cancer Cells

The ability of ZnD to effectively kill colorectal carcinoma cancer cells with acquired resistance to DOX studies in HCT116 DOX-resistant (HCT116 DR) cells was evaluated. DOX is a commercial chemotherapeutic that is used for the treatment of several cancer types that intercalates with DNA, RNA, and proteins to inhibit their synthesis. DOX interaction with DNA leads to an inhibition of topoisomerase-II activity, induction of single and double DNA strand breaks, and chromosomal aberrations [25,26]. Interestingly, the high cytotoxic and cytostatic potential of ZnD and the fact that it does not induce genotoxicity may indicate that its biological action is different from DOX, which might make ZnD suitable to inhibit the growth of DOX-resistant cancer cells.

ZnD showed a remarkable two-fold higher cytotoxicity in HCT116 DOX-resistant (HCT116 DR) cells. In fact, an IC50 at 48 h of 0.108 µM and of 0.215 µM were observed for HCT116 DR and for HCT116, respectively (see Table 1). These data clearly indicate the potential application of ZnD to tackle DOX-resistant colorectal cancer cells. Interestingly, HCT116 DR, which showed no decrease in cell viability up to 6 µM of DOX, revealed an increased sensitivity to ZnD compared to the HCT116 parental cell line.

A comparative proteomic analysis of the ZnD effect in HCT116 DR highlighted several molecular response profiles in clear agreement with the cell death mechanism identified earlier, such as an induction of cell death (via reactive oxygen species (ROS) induction and apoptosis) and cell cycle arrest as the main pathways involved (see Supplementary Material Section 4; Figure S4 and Table S1; STRING Analysis). Data show that these cancer cells have an efficient mechanism of ROS detoxification that allows them to survival under pro-oxidizing conditions [27], with higher activity for the DOX-resistant cells. The apoptotic and folding pathways that are activated upon ZnD treatment in HCT116 and HCT116 DR cells lead to a high loss of cell viability.

### 2.3. Nanovectorization of ZnD: Increasing Cytotoxicity

The altered vascular and lymph uptake functions induced by growing tumors may lead to the observed differential accumulation of nanoscale materials at the cancer site, in what is called the enhanced permeability and retention effect (EPR). As such, nanoscale vectors may passively make use of this EPR effect for the targeted delivery of chemotherapeutics [28,29,30]. This may be associated to active targeting via target selective moieties coupled to the nanoparticles, making these nanoconjugates valuable tools to direct chemotherapeutics selectively to the cells/tissue of interest. In the case of solid tumors, this active targeting potentiates selective uptake by cancer cells, thus avoiding drugs exerting their deleterious effect on healthy tissues. The active targeting of nanomedicines is usually achieved by formulations that incorporate moieties to selectively discern the molecular biomarkers of interest, such as the epidermal growth factor receptor (EGFR) [31]. Together, EPR and active targeting synergistically improve efficacy against tumor cells by improving the therapeutic effective doses.

The resistance of cancer cells to apoptosis has been associated to EGFR overexpression and to mutations in intracellular EGFR [32]. For example, EGFR overexpression has been observed in colorectal carcinoma and in approximately 40–90% of non-small cell lung cancer cases [32,33]. As such, EGFR would be the obvious choice as a target for the selective delivery of a nanoformulation, and it can be achieved via an anti-EGFR antibody, such as cetuximab. First, we used Western blot to confirm that EGFR was upregulated in HCT116 and HCT116 DR and in two non-small lung cancer cell lines, H1975 and A549 when compared to normal human primary fibroblasts (Supplementary Figure S5). HCT116 DR showed a 3.4-fold higher EGFR expression compared to fibroblasts. This confirmed that EGFR would be the ideal choice for targeting DOX-resistant cancer cells.

**Figure 4:**
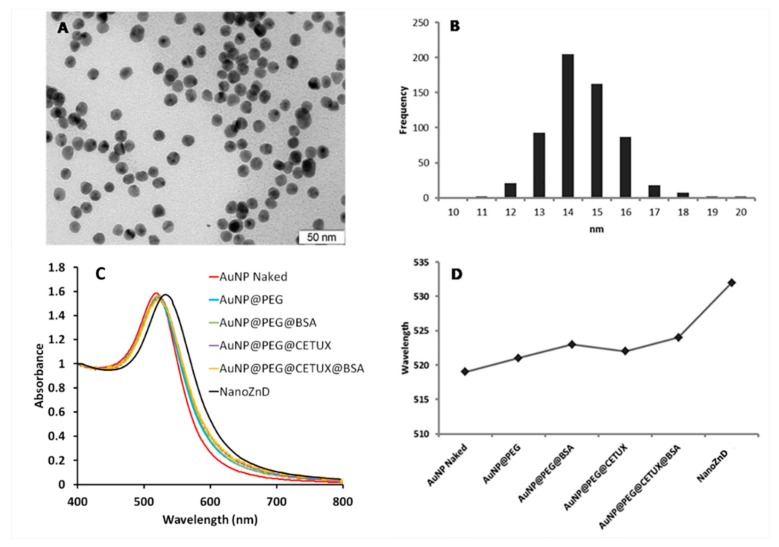
Physicochemical characterization of AuNP constructs. TEM image analysis of core naked AuNPs (scale bar, 50 nm) (**A**) evidencing an average diameter of 14 nm (**B**), UV/Vis spectra of AuNP constructs, (**C**) and surface plasmon resonance (SPR) peak variation upon each successful functionalization (**D**).

A multifunctional AuNP-based delivery system comprising ZnD and cetuximab (NanoZnD) was engineered to attempt the selective delivery of ZnD to cancer cells (see Figure 4, Table 2, and Supplementary Material). The AuNPs are first covered with polyethylene glycol (PEG) to increase stability, biocompatibility, and stealth properties, followed by the addition of the targeting moiety (cetuximab, here used solely as targeting for EGFR), and finally functionalization with bovine serum albumin (BSA) that will incorporate ZnD due to the strong affinity between the compound and albumin (Supplementary Material Figures S6 and S7).

To confirm that cetuximab leads to an increase in the internalization of the nanoconjugates, we assessed cell uptake by flow cytometry (Supplementary Figure S8). The variation of side scattering, which can be attributed to AuNPs (inside or at the membrane of cells), is higher for AuNPs@PEG@CETUX than for AuNPs@PEG, thus showing the effect of cetuximab in directing the nanoconjugates to the cells (Supplementary Figure S8).

**Figure 5:**
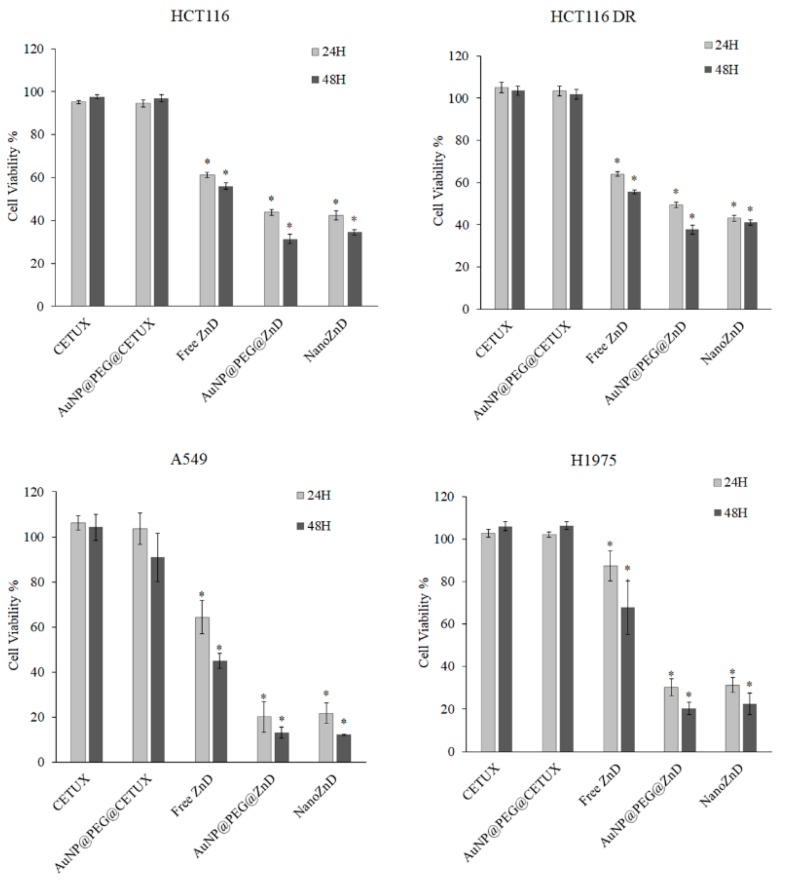
Cell viability assessment of the nanoconjugates in HCT116 and HCT116 DR cell lines after 24 and 48 h of incubation. Antiproliferative activities were assessed and compared to free ZnD (at its IC_50_ at 48 h) for each cell line (Table 1). Data are represented as mean ± SEM of at least three independent experiments. Cell viability was normalized to the control group without nanoparticle systems/compound. * *p* < 0.05 compared to control.

The in vitro efficacy of the nanoconjugates in cell lines expressing EGFR (HCT116, HCT116 DR, A549, and H1975) was evaluated (Figure 5 and Supplementary Figure S9, Table S2). The concentration of nanoconjugates used in all the assays were adjusted to deliver the same concentration of ZnD (IC_50_ at 48h), and the control formulations without compound were used at the same AuNP concentration. Data show a strong reduction in cell viability (~20–50%) for cells challenged with nanoconjugates when compared to free ZnD (Figure 5 and Table S2), demonstrating the in vitro potential of ZnD nanoformulations. This is particularly clear against DOX-resistant colorectal cancer cells and EGFR-overexpressing cell lines. As previously demonstrated for similar nanovectorization systems [6,34,35], the nanovector alone (without ZnD) does not impair cell viability (Figure 5). No cytotoxic effect was expected for AuNP@PEG@CETUX since, in the nanoformulation, cetuximab is at nanomolar concentration, which is largely below any reported cellular toxicity, and herein is used solely as a targeting moiety (Figure 5).

The nanoconjugates showed only a minor effect on fibroblasts, as previously shown for the free ZnD (Table 1). The nanoconjugates showed a similar trend of relative cytotoxicity to that of the free ZnD with HCT116 DR > HCT116 > H1975 > Fibroblasts ~ A549, but with a higher cytotoxic effect (Supplementary Material Figure S10).

**Figure 6:**
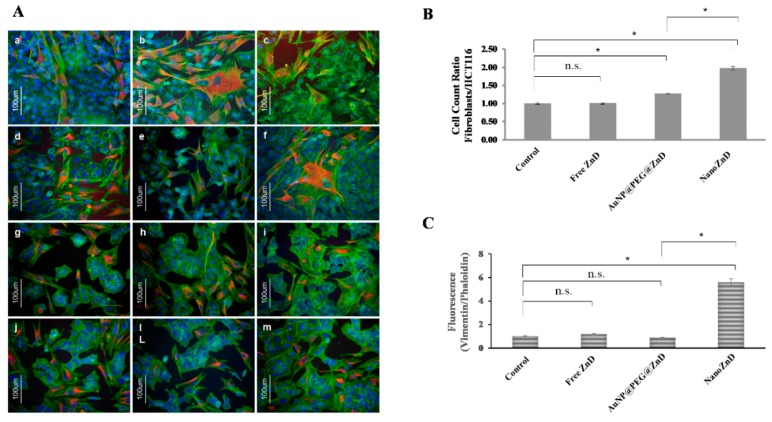
(**A**) Immunofluorescent images of the HCT116 and fibroblasts primary cell co-culture (vimentin stained in red). Co-cultures were incubated with fresh growth medium (**a**–**c**), ZnD at its respective IC_50_ at 48 h (Table 1) (**d**–**f**), AuNP@PEG@ZnD nanoconjugate (**g**–**i**) and NanoZnD (**j**,**l**,**m**) for an incubation period of 6 h. Immunofluorescent images were acquired with a Zeiss Axioplan 2 Imaging Microscope and a Nikon DXM1200F digital camera. (**B**) Ratio Fibroblasts/HCT116. Cell count was performed using ImageJ Software. (**C**) Average corrected total cell fluorescence (CTCF) ratio, vimentin/phalloidin, of the co-cultures exposed to fresh growth medium, ZnD (IC_50_), and with nanoparticle constructs functionalized with ZnD, AuNP@PEG@ZnD, and NanoZnD. Data represents the mean of the fold variation compared to the control sample without AuNPs (fresh growth medium). Data represents the mean ± SEM of at least three different images; * *p* < 0.05 compared to control. n.s.—no statistical difference.

Regardless of the targeting moiety being present, the results show similar in vitro efficacy of both nanoformulations to kill cancer cells in the tested models (HCT116, HCT116 DR, and H1975), i.e., no apparent enhancement of efficacy for NanoZnD. Nevertheless, NanoZnD ought to show increased selectivity toward cancer cells compared to normal fibroblasts, thus minimizing side effects. In this regard, we evaluated the selective effect on cancer cells by monitoring the ratio of fibroblasts/HCT116 cells in the co-cultures of both cells challenged with free ZnD or the target and non-targeted nanoconjugates, as shown in Figure 6. Immunofluorescent images of cells stained with vimentin (stains mesenchymal cells such as fibroblasts, thus providing differential cell staining) and phalloidin (stains all cells) show that all formulations induced a decrease in cellular density, which is more evident when challenged with NanoZnD nanoconjugates (Figure 6A). More interestingly, Figure 6B shows that the NanoZnD nanoformulation showed the highest ratio of cancer cell death (decreasing number of HCT116 cells per fibroblast), thus evidencing the effect of the selective targeting of cetuximab. Indeed, cell counts on the immunofluorescent images of co-cultures showed that twice as many HCT116 cells die per fibroblast. This effect is more pronounced for the cetuximab-functionalized nanoconjugates than for the AuNP@PEG@BSA@ZnD counterpart. To further corroborate these analyses, we quantified the corrected total cell fluorescence (CTCF) ratio vimentin/phalloidin of each sample (Figure 6B). Co-cultures exposed to free ZnD and AuNP@PEG@ZnD do not exhibit significant changes to the CTCF ratio. Conversely, NanoZnD induces a 5.6-fold increase to the vimentin/phalloidin ratio, which illustrates the efficacy of the selective targeting by NanoZnD (Figure 6C) and the role of cetuximab for potentiating the delivery of ZnD to cancer cells (reducing effects to normal cells).

### 2.4. In Vivo Assays

**Figure 7:**
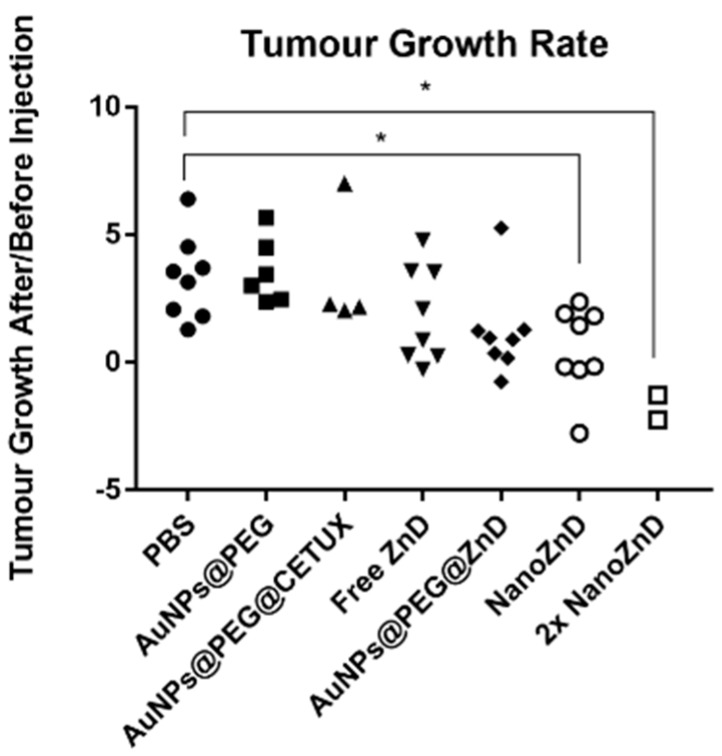
Tumor growth rate in mice model. Ratio of tumor growth rate before (T0) and after injection of formulation (T1 or T2). Each point represents one injection. Values greater than one represent an increase in the tumor growth rate after injection. Values between one and zero represent a decrease in the tumor growth rate. Negative values indicate not only decrease a in the tumor growth rate, but also tumor remission (* *p* < 0.05).

**Figure 8:**
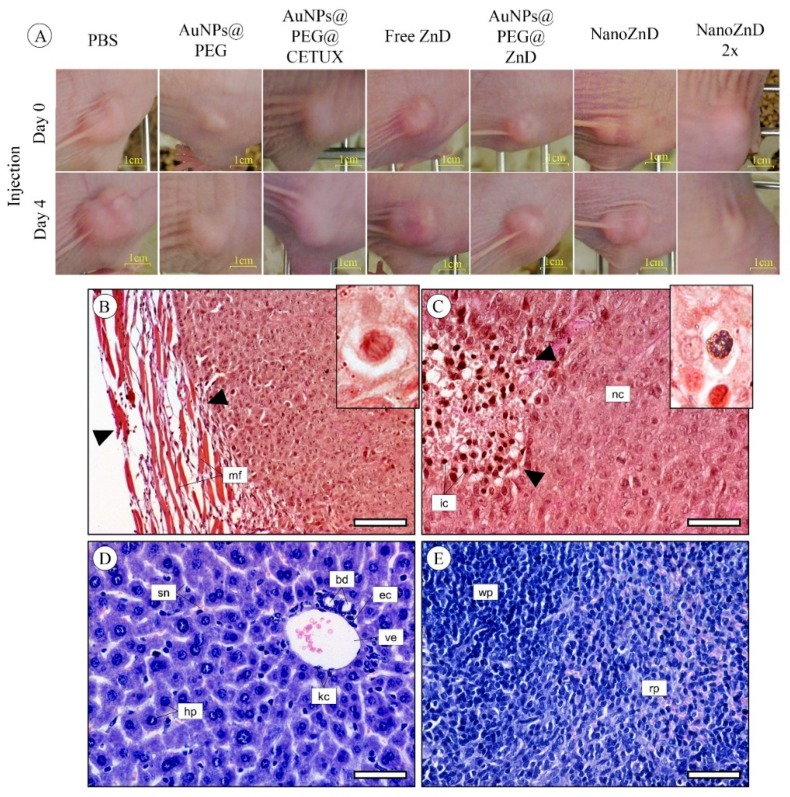
(**A**) Images of mice tumor xenografts on the day of injection of each formulation and four days later. Example of micrographs of representative histological sections of tumor, liver, and spleen from tested mice. (**B**) Mixed tumor border configuration, showing part of the fibrous encapsulation (between arrowheads), formed by connective tissue and muscle fibers (mf). Inset: Detail of a mitosis in tumor cells. (**C**) Tumor presenting a compact mass of neoplastic cells (nc) and foci of infiltrating inflammatory cells (ic), within which are found debris of necrotic cells (arrowheads). Inset: Detail of a small metallic agglomerate inside a macrophage found in a tumor from a mouse injected with gold nanoparticles. (**D**) Representative micrograph of the hepatic tissue of mice, showing the normal architecture of the organ, i.e., with regular-sized hepatocytes (hp) arranged in trabeculae. bd: Bile duct, ec: Endothelial nucleus cell, kc: Kupffer cells, sn: Sinusoids, ve: Venule. (**E**) Representative micrograph of the spleen of tested mice. Regardless of experimental treatment, no histopathological alterations were found in the organ. rp: Red pulp, wp: White pulp. (**B**) and (**C**), tetrachrome (TC) stain. (**D**) and (**E**), Gill’s Alum Hematoxylin and counterstained with alcoholic Eosin Y (H&E), Insets, neutral red. Scale bars: (**B**): 100 μm, (**C**–**E**): 50 μm.

Upon successful demonstration of the in vitro selective targeting capability and cytotoxic potential of NanoZnD, the in vivo efficacy of these nanoformulations was evaluated on HCT116 DR-derived xenografts. Tumors were allowed to grow until ~10 mm^3^ and treatments were performed by two intravenous tail injections separated by four days using AuNP@PEG, AuNP@PEG, AuNP@PEG@CETUX, AuNP@PEG@ZnD, and NanoZnD (and PBS as control), and animals were sacrificed five days after the second administration. Tumors, spleen, and liver were extracted, and divided for inductively coupled plasma atomic emission spectrometry (ICP-AES) and histology analysis. Two days after each injection, the tumor volume was evaluated. For PBS, AuNPs@PEG, and AuNPs@PEG@CETUX, we did not observe any reduction in the tumor growth rate prior to injection versus after injection (Figure 7 and Figure 8A). For free ZnD, there was a 26% reduction in the tumor growth rate. For the non-targeted nanoformulation (AuNPs@PEG@ZnD), the reduction was more pronounced (76%). The targeted nanoformulation, NanoZnD, reduced tumor growth in all treated mice when compared to controls. This shows the in vivo efficacy of the nanoformulation to selectively tackle DOX-resistant cancer cells.

We further assessed the potency of the nanoformulation by testing the effect of a single dosage administration at twice the concentration of NanoZnD (total dose per animal remaining the same). In this case, the tumor growth rate reduced by an average of 176%. This shows that there is a chance for improving efficacy by escalating the dose without increasing toxicity, since neither accumulation of the vehicle for the remaining formulations nor toxicity effects were perceptible. In fact, ICP-AES quantification showed the presence of gold only in the tumor samples with no detectable gold in the spleen or liver (results not shown).

Overall, the subcutaneous tumors presented a similar histological morphology between controls and treatments. Most tumors showed a mixed border configuration, which is possible due to fibrous encapsulation, and in some cases, the mass was found to be aggressively intruding the surrounding mouse tissue (Figure 8B). The neoplastic cells were actively proliferating independently of treatment, as indicated by a high frequency of cells in mitosis (Figure 8B—Inset), which is indicative of the continuous development of the tumor. The central area of the tumor was invariably characterized by a massive infiltration of inflammatory cells and lymphoid tissue (Figure 8C). Small metallic agglomerates were detected—in the histological preparations—inside a macrophage in tumors from mice injected with gold nanoparticles (Figure 8C—Inset). Together with the ICP-AES data, these results show that the nanoformulations accumulate almost entirely at the tumor site, which supports the idea of direct targeting to the tumor. No visible histopathological alterations were found in the liver (Figure 8D) and spleen (Figure 8E) of mice subjected to any of the experimental treatments, including controls. Opposite to what was found in tumors, no metallic deposits consistent with AuNP agglomerates were detected in the liver and spleen of tested mice. Together, these data demonstrate the targeting potential for the nanoformulation coupled to the anti-tumor activity of the compound, without accumulation in healthy tissues and organs, and without any observable toxic side effects.

## 3. Materials and Methods

All reagents were of analytical grade and purchased from Sigma-Aldrich (St. Louis, MD, USA). The Zn(II) coordination compound ([Zn(DION)_2_]Cl_2_) ZnD was synthesized and characterized as previously described [1]. Millipore^®^ (Burlington, Massachusetts, USA) water was used for the preparation of all aqueous solutions. Cetuximab (Erbitux^®^, Amsterdam, Netherlands) was provided by Merck Serono (Darmstadt, Germany). All other reagents were of analytical grade.

### 3.1. Cell Culture

HCT116 colorectal carcinoma, A549 lung adenocarcinoma, and primary human fibroblasts obtained from ATCC (www.ATCC.org, Chicago, IL, USA) were grown in Dulbecco’s modified Eagle medium (DMEM; Invitrogen, Carlsbad, CA, USA) and supplemented with 10% (*w*/*v*) fetal bovine serum (FBS, Invitrogen) and 1% (*w*/*v*) antibiotic/antimycotic (Invitrogen). NCI-H1975 [H-1975, H1975] human lung adenocarcinoma cells were grown in RPMI (Roswell Park Memorial Institute) 1640 medium (RPMI; Invitrogen, Carlsbad, CA, USA) supplemented with 10% (*w*/*v*) FBS and 1% (*w*/*v*) antibiotic/antimycotic solution (Invitrogen). Cells were maintained in 75 cm^2^ culture flasks (VWR) at 37 °C in a 99% (*v*/*v*) humidified atmosphere of 5% (*v*/*v*) CO_2_ (CO_2_ Incubator Leec, Nottingham, UK). Primary human fibroblasts were cultured as previously described [4]. HCT116 Doxorubicin-resistant (HCT116 DR) cells were derived from HCT116 by successive culturing cells with increasing concentrations of DOX over each passage up to a final concentration of 3.6 µM DOX [36].

### 3.2. Cytotoxicity

Cell viability was assessed by the [3-(4,5-dimethylthiazol-2-yl)-5-(3-carboxymethoxyphenyl)-2-(4-sulfophenyl)-2H-tetrazolium] (MTS) assay as previously described [1,2,3,4]. Briefly, fibroblasts, HCT116, HCT116 DR, A549, and H1975 cells were seeded at a density of 7.5 × 10^3^ cells/well in 96-well plates and grown for 24 h prior to incubation for 24 and 48 h in fresh medium with and without the nanoconjugates or free ZnD. The concentration of each nanoconjugate was determined to match the equivalent number of molecules of free ZnD (IC_50_). The IC_50_ was determined (at 48 h) for the free ZnD for all cell lines. The percentage of cell viability for each concentration was determined considering at least three independent biological assays.

### 3.3. Cell Death Mechanism

#### 3.3.1. Hoechst 33258 Staining

HCT116 cells were plated in 35-mm dishes at 1.5 × 10^5^ cells/dish. Culture medium was removed 24 h after plating and replaced with 2 mL of fresh medium containing either free ZnD (IC_50_) or water (vehicle control). Cells were stained after an incubation period of 48 h with Hoechst as previously described [1].

#### 3.3.2. Quantification of Cell Death by Flow Cytometry

HCT116 cells were seeded into 35-mm dishes at 1.5 × 10^5^ cells/dish. Culture medium was removed 24 h after platting and replaced with 2 mL of fresh medium containing ZnD (IC_50_) or water (vehicle control). Cells were incubated in the presence of ZnD for 48 h and then stained with propidium iodide (PI) and fluorescein isothiocyanate (FITC) labeled annexin V according to the manufacturer’s instructions (Annexin V-FITC Apoptosis Detection Kit, Invitrogen, Carlsbad, USA) [1,4].

#### 3.3.3. Caspase-3/-7 Activity

HCT116 cells were plated at 7.5 × 10^3^ cells/well in a black opaque 96-well microplate (Corning, New York, USA). Media was removed 24 h after plating and replaced with fresh media containing ZnD (IC_50_) or water (vehicle control). The blank was made with culture medium without cells. Cells were incubated for 48 h at 37 °C and 5% (*v*/*v*) CO_2_. Caspase-3/-7 combined activity was quantified by using the Apo-ONE^®^ Homogeneous Caspase-3/7 Assay (Promega, Madison, Wisconsin, USA) according to the manufacturer’s instructions and as previously described [2,3].

#### 3.3.4. Mitochondrial Membrane Potential

To evaluate the mitochondrial membrane potential, the fluorescent dye 5,5,6,6-tetrachloro-1,1,3,3 tetraethylbenzimidazolylcarbocyanine iodide (JC-1) (Abnova Corporation, Walnut, CA, USA) was used. HCT116 cells were seeded with a cell density of 7.5 × 10^4^ cells/well in a 6-well plate for 24 h. After exposure to the IC_50_ for DOX (0.42 µM) and ZnD for 48 h, JC-1 was added to the medium for 20 min. The fluorescence of the JC-1 monomer and dimmer was analyzed on an Attune^®^ Acoustic Focusing Flow Cytometer (Life Technologies, Carlsbad, CA, USA) and Attune^®^ Cytometric software (Life Technologies).

### 3.4. Analysis of Cell Cycle

HCT116 cells were seeded in 25-cm^2^ culture flasks with a density of 7.5 × 10^3^ cells/well and were synchronized in an early S-phase by a double thymidine block, as previously described.^25^ Cells were released from the second block by substituting the medium with 2 mM of thymidine (Sigma-Aldrich) for fresh medium without thymidine and either with ZnD (IC_50_) or water (vehicle control). After incubation periods of 6 h and 12 h at 37 °C and 5% (*v*/*v*) CO_2_, media were removed and cells trypsinized, and cell cycle analysis proceeded as described in [2,3]. DNA content was analyzed on an Attune^®^ Acoustic Focusing Flow Cytometer (Life Technologies) and Attune^®^ Cytometric software (Life Technologies).

### 3.5. Chromosomal DNA Alterations

#### 3.5.1. Chromosomal Aberrations

For chromosomal aberration analysis, 1 × 10^6^ V79 cells were treated with 1.5 µM mitomycin C and ZnD (IC_50_) for 16 h in 37 °C and 5% CO_2_, and the protocol proceeded as described in [2,3].

#### 3.5.2. Genotoxicity Assessment

Genotoxic effects were assessed by a quantification of DNA strand breakage, through the alkaline version of comet-assay and frequency of nuclear abnormalities as previously described [3]. HCT116 cells were plated at 1 × 10^6^ cells/dish in 35-mm dishes and allowed to attach for 24 h at 37 °C and 5% (*v*/*v*) CO_2_. Culture medium was removed and replaced with 2 mL of fresh medium containing either ZnD (IC_50_) or water (vehicle control) for 12 and 18 h, at 37 °C and 5% (*v*/*v*) CO_2_. Hydrogen peroxide (H_2_O_2_) at 0.05% (*v*/*v*) for 30 min at room temperature was used as positive control. The percentage of DNA in the tail was used as measure of the total DNA strand-breakage.

### 3.6. Interaction Studies of ZnD with Albumin

Bovine serum albumin (BSA) stock solution at 50 µM was prepared by gently dissolving the protein in phosphate buffer pH 7.0 with 0.15 M NaCl, which was gently swirled for 45 min to allow the protein to hydrate and fully dissolve. The concentration of BSA stock solution was determined by UV spectrophotometry using the molar extinction coefficient at 280 nm (43,824 M^−1^cm^−1^). Spectroscopic measurements were carried out on individually prepared samples to ensure the same pre-incubation time at (37.0 ± 0.5) °C in each essay. BSA concentration was kept constant at 0.13 µM, while the concentration of the complex ranged from 0 to 375 µM. Samples were incubated at 37 °C for 1 h.

### 3.7. Spectroscopic Measurements

UV-visible measurements of a 50-µM ZnD solution were prepared in RPMI biological medium, but no phenol red (Gibco™, Carlsbad, CA, USA) was carried out immediately after their preparation or after a 24 or 48-h incubation period at 37 °C. UV-visible absorption spectra were recorded at room temperature on a UV-VIS spectrophotometer (UVmini 1240, Shimadzu, Kyoto, Japan) in the range of 200–800 nm with a 1-cm path quartz Suprasil^®^ cuvette (Hellma^®^ Analytics, Jena, Germany). For nanoparticles’ characterization, UV-visible absorption spectra were recorded at room temperature on a UV-Vis spectrophotometer (UVmini 1240, Shimadzu, Kyoto, Japan) in the range of 230–800 nm with 1-cm path quartz Suprasil^®^ cuvettes as described previously [6]. Fluorescence measurements were carried out on a Cary Eclipse Fluorescence spectrophotometer (Agilent Technologies, Santa Clara, CA, USA) at room temperature as described previously [6].

### 3.8. Gold Nanoparticles Synthesis and Assembly of Au Nanoconjugates

AuNPs (AuNP@citrate refer as AuNPs hereafter) were synthesized by the citrate reduction method described by Lee and Meisel and characterized by UV-Vis spectroscopy, transmission electron microscopy (TEM), and dynamic light scattering (DLS) [6]. AuNPs were subsequently functionalization with polyethylene glycol (PEG) as previously described [6]. AuNP@PEG were functionalized with bovine serum albumin (BSA) (Sigma-Aldrich, MW 66,120 kDa) (AuNP@PEG@BSA) via the EDC/NHS coupling reaction^6^. AuNP@PEG were also functionalized with cetuximab (Erbitux^®^, Merck Serono) (AuNP@PEG@CETUX) and with the same BSA concentration 1 h prior to antibody addition (AuNP@PEG@CETUX@BSA) under the previously described conditions.^6^ Cetuximab antibody was added to reaction mix at a final concentration of 3 μg/μL. Following 1 h of incubation with cetuximab, BSA was added to the reaction mix at a final concentration of 10 µg/mL, and the total volume was allowed to react for 16 h at RT. The total amount of conjugated BSA and/or cetuximab was estimated by subtracting the amount of removed protein (during the centrifugation steps) to the total protein amount added in the first place for functionalization purposes. For conjugation with ZnD, 6-nM AuNP@PEG@BSA and 6-nM AuNP@PEG@CETUX@BSA were mixed separately with 50 μM of ZnD (in water) and incubated for 1 h at 4 ºC to allow for compound incorporation into the nanoparticle conjugates to obtain AuNP@PEG@BSA@ZnD and AuNP@PEG@CETUX@BSA@ZnD (NanoZnD), respectively. After this period, solutions were centrifuged at 14,000× *g* for 30 min at 4 °C to remove excess of ZnD. The supernatants were recovered and analyzed by a UV-Vis spectrophotometer and inductively coupled plasma atomic emission spectrometry (ICP-AES) to assess ZnD content, and the number of compound molecules per nanoparticle calculated.

### 3.9. Au Nanoconjugates Characterization

Au nanoconjugates were characterized by UV-Vis, DLS, and TEM as previously described [6].

### 3.10. AuNPs—Cell Interaction (Flow Cytometry)

Cell uptake was assessed by flow cytometry analysis as previously described^6^. Nanoformulations exposure (AuNP@PEG and AuNP@PEG@CETUX) was allowed to react for a total period of 6 h under the conditions mentioned to ensure AuNP uptake. Following DMEM removal, cells were extracted by trypsinization and stored in 2-mL microfuge tubes. Cell samples were analyzed by flow cytometry as described above through the acquisition of at least 10,000 events for each experimental condition.

### 3.11. Co-Cultures—Selectivity Assay

HCT116 cells and primary human fibroblasts were seeded at a cell density of 1 × 10^4^ cell/well (1:1 ratio) over a cover slip in a 24-well plate well for 24 h, and then incubated with nanoconjugates for 6 h. Cells were fixed with 4% (*v*/*v*) paraformaldehyde (10 min, room temperature) and permeabilized with 0.1% (*v*/*v*) Triton X-100 for 5 min. Cells were double stained with anti-vimentin 1:200 (Sigma-Aldrich) for 1 h, which stains cells of mesenchymal origin (fibroblasts) and not epithelial cells (HCT116), anti-mouse TRITC 1:64 (Sigma-Aldrich) for 30 min, and with Phalloidin 488 Alexa Fluor 3:200 (Invitrogen, Life Technologies, USA) for 20 min and analyzed as previously described.^6^ Cell number count and the fluorescence quantification of co-culture’s immunofluorescent images were performed for exposition to: (i) fresh growth medium (control), (ii) ZnD at its respective IC_50_, and (iii) AuNP@PEG@BSA@ZnD and NanoZnD for 6 h using at least three different images. Cell number count and the corrected total cell fluorescence (CTCF) ratio of vimentin/phalloidin was calculated through ImageJ software.

### 3.12. Animal Model Assays

Five-week-old male BALB/c nude mice (30 animals) were purchased from Charles River, France. All animal experiments were carried out with the permission of the Portuguese Authority (Direcção Geral de Alimentação e Veterinária), and the study was approved by the Local Animal Ethical Committee (Comissão de Ética Experimentação Animal da Faculdade de Farmácia, Universidade de Lisboa), and in accordance with the Declaration of Helsinki, the EEC Directive (2010/63/UE, 22 of September 2010), and Portuguese law (DL 113/2013, Despacho no 2880/2015, 20 of March 2015), and all following legislation for the humane care of animals in research. Animals were kept under sterilized conditions using an Air Handling Easy Flow Ventilation Unit with single-sided racks and Blue Line NEXT individually ventilated cages (Tecniplast, West Chester, Philadelphia, USA). Animals were fed and maintained as previously described [6].

Mice were randomly distributed in six groups with five animals per group for each experimental condition. HCT116 DR-derived xenografts were induced by s.c. administration of 1 × 10^6^ cells at the back hip of mice. Tumors were allowed to grow until an approximate size of 10 mm^3^, and treatments were performed with two i.v. injection in the tail vein separated by four days. The administrations were performed according with the following groups: PBS 1X, free ZnD, AuNP@PEG, AuNP@PEG@CETUX, AuNP@PEG@ZnD, and NanoZnD, with 5.6 ng/kg of ZnD per mice per injection (free or in the AuNP conjugates). Tumor volume was determined three times a week by measuring with calipers in two dimensions and by image analysis. Tumor volume (V) was estimated assuming an ellipsoid conformation where the height was estimated according to the tumor proportions at the day of sacrifice. Mice were monitored routinely for physical status. Mice were sacrificed five days after the last treatment. At the end of the experience, mice were anesthetized with isoflurane (Isoflo, Esteve Farma, Carnaxide, Portugal), animals were sacrificed by neck hyperextension and organs of interest (spleen and livers), and tumors were extracted weighted, washed with cold 0.154 M KCl to remove the excess blood, and divided in two pieces: One for an ICP-MS quantification of gold and the other for histological studies. The tumor growth rate was calculated for individual animals by determining the linear slope of tumor volume growth, before injection or after injection. The average of the tumor growth rates was determined for each group before and after injection.

### 3.13. Histological Analysis

Samples of liver, spleen, and tumor were carefully excised fresh and immediately fixed in 10% (*v*/*v*) neutral-buffered formalin solution (Sigma-Aldrich) for 24 h. After fixation, the samples were processed as described previously. Liver and spleen sections were stained with Gill’s Alum Hematoxylin and counterstained with alcoholic Eosin Y (H&E). Tumors were stained with a tetrachrome stain (TC) for fibers and nuclei that include Alcian Blue, Weigert’s Iron Hematoxylin, and van Gieson’s dye [37], similarly to our previous work [6]. Sections of all samples were stained with neutral red to enhance contrast and thus detect metallic gold deposits with a purplish color. Finishing the staining, all the sections were dehydrated in ethanol, cleared in xylene, and mounted with DPX Mountant (Sigma-Aldrich). Histological analyses were made with a DM 2500 LED model microscope equipped with an MC 190 HD camera (both from Leica).

### 3.14. Statistical Analysis

Statistical analysis was performed for all assays using GraphPad Prism v6.01. (GraphPad Software, San Diego, CA, USA) using the Mann–Whitney test. All data were expressed as mean ± SEM from at least three independent experiments.

For in vivo analysis, groups were compared using the Kruskal–Wallis test. Statistical significance was considered when *p*-value < 0.05.

## 4. Conclusions

The surge in drug resistance in cancer has prompted the development of alternative strategies to circumvent the diminished response of tumors to standard chemotherapy. The impact of novel compounds in cancer chemotherapy is often challenged by stability and solubility issues and, more importantly, a lack of selectivity to cancer cells that induce undesirable deleterious effects to healthy cells and tissues. Here, we presented a nanoformulation for the targeted delivery of a powerful anti-tumor water-soluble Zn(II) coordination compound ([Zn(DION)_2_]Cl_2_) (ZnD) toward colorectal carcinoma cells. ZnD high cytotoxic activity was associated to an increased cell death by apoptosis and the induction of cell cycle arrest in the S phase without observable genotoxicity. This could be a valuable cue to circumvent tumor cells showing resistance to doxorubicin. The gold nanoparticle-based nanoformulation herein characterized was used to deliver ZnD selectively to colorectal cancer cells via active targeting mediated by cetuximab: NanoZnD. NanoZnD showed and enhanced in vitro antiproliferative potential against sensitive and DOX-resistant colorectal carcinoma cancer cell lines. We demonstrate that cetuximab provides for selective targeting to cancer cells and an increased uptake of ZnD, resulting in selective cancer cell death. The promising anticancer capability of this nanoformulation was further demonstrated in vivo, where it reduced tumor growth without any deleterious effect to healthy tissues. What is more, this nanoformulation was capable of effectively reducing DOX-resistant colorectal cancer cells in a single dose without systemic toxicity.

## Figures and Tables

**Table 1:** Relative IC_50_ (μM) of ZnD in HCT116, HCT116 DR, A549, and H1975 human cell lines at 48 h. Values shown are relative to the mean of three independent assays and the errors are correspondent to SEM.

Human Cell Line	Relative IC_50_ ± SEM
HCT116	0.215 ± 0.01
HCT116 DR	0.108 ± 0.01
A549	0.714 ± 0.09
H1975	0.355 ± 0.04
Fibroblasts	0.600 ± 0.13

**Table 2:** Nanoconjugate functionalization moieties and hydrodynamic size determination by dynamic light scattering (DLS). The different functional moieties and DLS size are discriminated (represented as means ± SEM of at least three independent experiments). The ratio of protein molecules and/or ZnD per nanoparticle in each nanoformulation is indicated.

AuNP Conjugate	Proteins per AuNP	ZnD per AuNP	DLS (nm)
AuNPs citrate	-	-	15.3 ± 0.2
AuNP@PEG	-	-	18.6 ± 0.3
AuNP@PEG@BSA	7.0 ± 0.5	-	27.4 ± 0.4
AuNP@PEG@CETUX	1.6 ± 0.2	-	78.3 ± 0.7
AuNP@PEG@CETUX@BSA	6.9 ± 0.5	-	110.4 ± 0.8
AuNP@PEG@BSA@ZnD	7.0 ± 0.5	402 ± 32	118.7 ± 0.9
NanoZnD	6.9 ± 0.5	438 ± 19	126.3 ± 0.9

## Supplementary Materials

Supplementary materials can be found at https://www.mdpi.com/1422-0067/20/21/5510/s1.

## Abbreviations

AuNPs	Gold Nanoparticles
BSA	Bovine Serum Albumin
DLS	Dynamic Light Scattering
DMEM	Dulbecco’s Modified Eagle Medium
DOX	Doxorubicin
EGFR	Epidermal Growth Factor Receptor
EPR	Enhanced Permeability and Retention Effect
FITC	Fluorescein Isothiocyanate
FBS	Fetal Bovine Serum
HCT116 DR	HCT116 Doxorubicin-Resistant
ICP-AES	Inductively Coupled Plasma Atomic Emission Spectrometry
JC-1	5,5,6,6-tetrachloro-1,1,3,3 tetraethylbenzimidazolylcarbocyanine iodide
MTS	3-(4,5-dimethylthiazol-2-yl)-5-(3-carboxymethoxyphenyl)-2-(4-sulfophenyl)-2H-tetrazolium
PEG	Polyethylene Glycol
PI	Propidium Iodide
RPMI	Roswell Park Memorial Institute
SPR	Surface Plasmon Resonance
TEM	Transmission Electron Microscopy
TC	Tetrachrome Stain
ZnD	DION - 1,10-phenanthroline-5,6-dione
