# Supplementary material for: Targeting Cancer Resistance via Multifunctional Gold Nanoparticles
Source: Int J Mol Sci. 2019 Nov 5;20(21):5510. doi: 10.3390/ijms20215510 (PMC6861975; doi:10.3390/ijms20215510)
Supplement: Supplementary file 1 [file ijms-20-05510-s001.pdf]

Supplementary Material

1. ZnD Stability

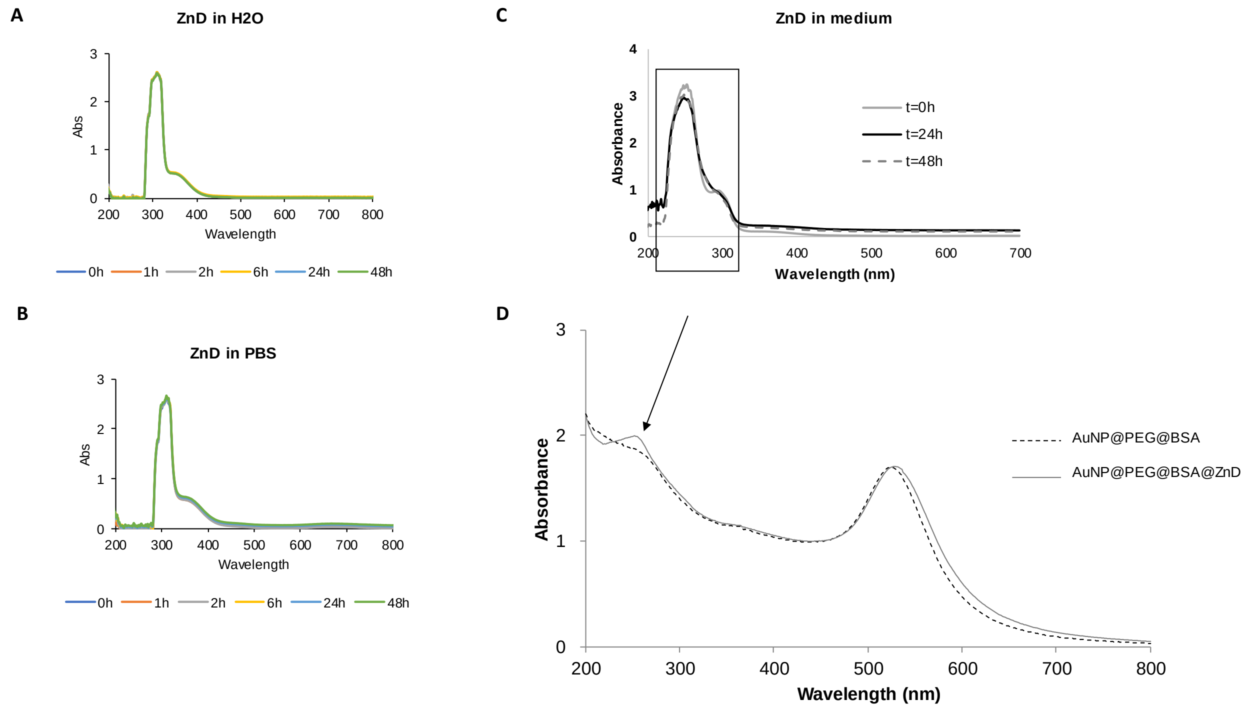

Figure S1. UV-Vis spectra of a 50 µM solution of ZnD in water (**A**), PBS (**B**) and in RPMI biological medium (**C**) over a period of 48h. The spectra of AuNP@PEG@BSA and AuNP@PEG@BSA@ZnD was also assessed after 48 h in RPMI biological medium (**D**); the ZnD maximum peak at 254 nm is indicated by an arrow in the AuNP@PEG@BSA@ZnD nanoconjugate; in both nanoformulations the nanoparticle SPR peak is also identified. The mesurements were performed in a UV–VIS spectrophotometer (UVmini 1240, Shimadzu, Germany) in the range 200–700 nm with 1 cm path quartz Suprasil® cuvette (Hellma® Analytics, Germany).

2. Mitochondrial membrane potential

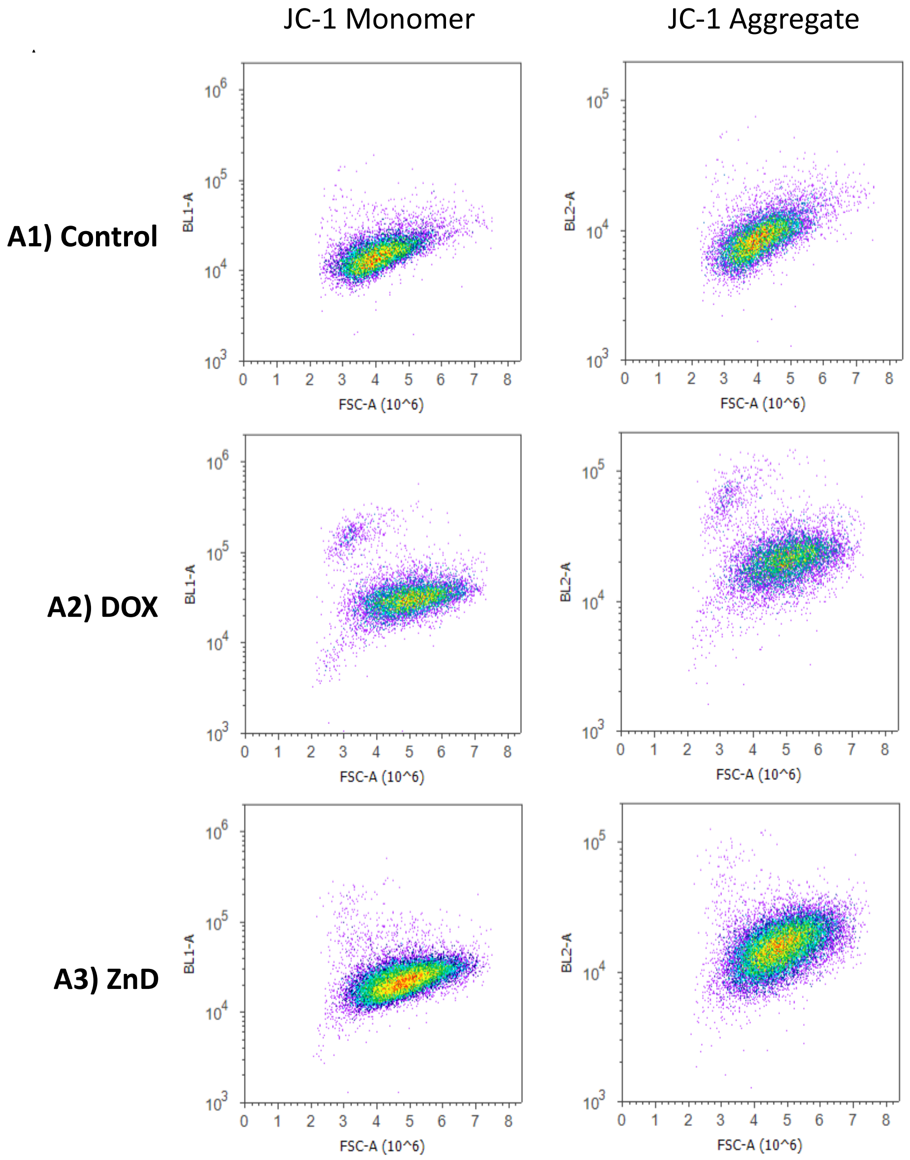

Figure S2. Mitochondrial membrane potential of HCT116 cells treated with ZnD. The fluorescence of JC-1 monomer, corresponding to green fluorescence, was acquired using filter BL1 (excitation and emission range wavelengths of 488 nm and 515-545 nm, respectively). The fluorescence of JC-1 aggregate, corresponding to red fluorescence, was acquired using filter BL2 (excitation and emission range wavelengths of 488 nm and 561-587 nm, respectively). Density plots of HCT116 cells incubated for 48 h with DMEM supplemented with 10% (*v*/*v*) fetal bovine serum and A1) control, A2) 0.42 μM DOX and A3) IC50 at 48 h ZnD, stained with JC-1 and fluorescence of cells was quantified using an Attune acoustic focusing cytometer (ThermoFisher Scientific).

3. Genotoxic potential of ZnD

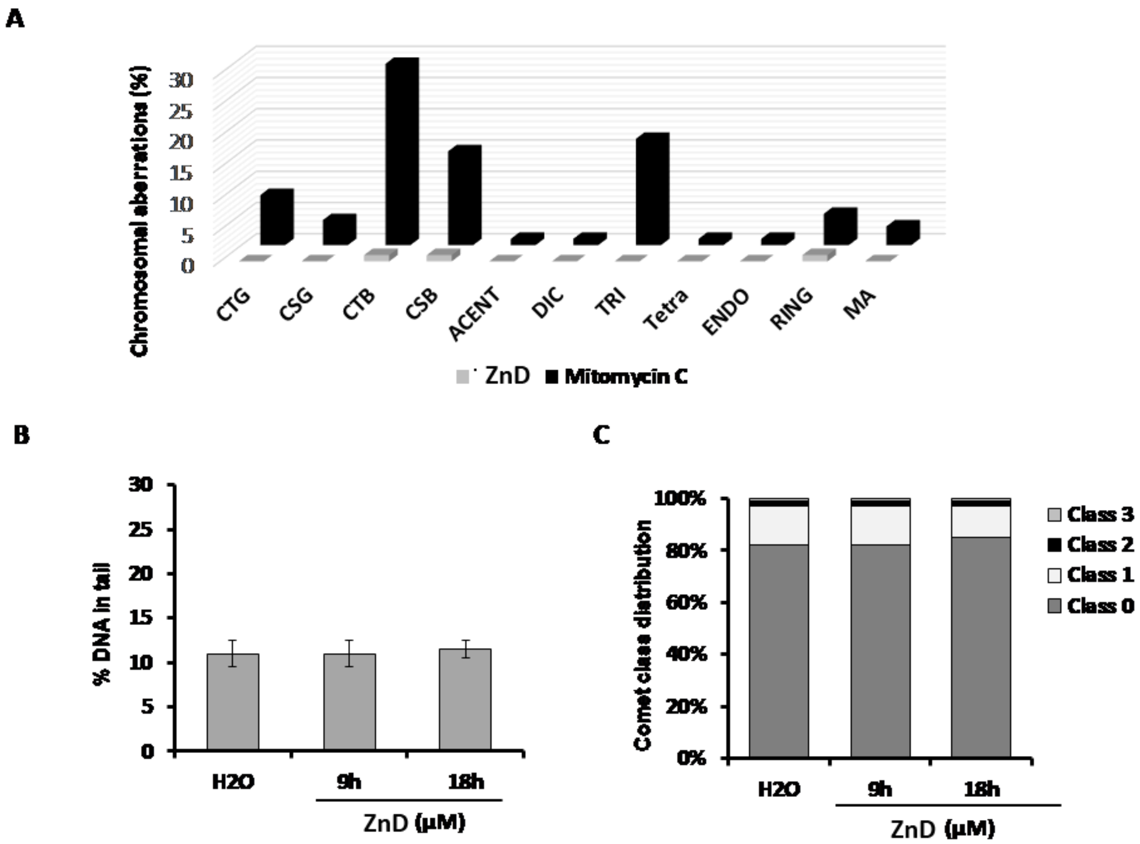

Figure S3. Chromosomal DNA alterations. A. Induction of chromosomal aberrations in V79 cells by 1.5 μM mitomycin C (black bars) and 0.217 μM of ZnD (light grey) exposure during 18 h. CTG, chromatid gap; CSG, chromosome gap; CTB, chromatid break; CSB, chromosome break; ACENT, acentric chromosome; DIC, dicentric chromosome; TRI, triradial chromosome; TETRA, tetraradial chromosome; ENDO, endoreduplication; RING, ring chromosome; MA, cells with multiple chromosomal aberrations. B. DNA percentage in the tail (% DNA in tail) and C. the comet class distribution in HCT116 cells treated with 0.217 μM ZnD for 9 and 18 h and H_2O (vehicle control) during 18 h.

4. Comparative Proteomic analysis

Preparation of protein extracts and two-dimensional electrophoresis

HCT116 DR cells were seeded in 75 cm^2 flask at a density of 4 x 10^6 cells/flask. Culture medium was removed after 24 h and replaced with fresh medium containing either 0.108 μM of ZnD (IC_{50} – Table 1) or water and cells were incubated for 48 h. Protein extracts and two-dimensional electrophoresis were prepared as previously described [1]. All 2-DE gel images were digitalized in an Image Scanner II (GE Healthcare) and analysed with Melanie 7.0 Software (GeneBio, Geneva, Switzerland) according to [1]. For identification of proteins of interest, protein spots were manually excised from the gel and identified in the UniMS Mass Spectrometry Unit, ITQB/IBET (Oeiras, Portugal) using Peptide Mass fingerprint. The groups of altered proteins between conditions were analysed in STRING (Search tool for the retrieval of interacting genes/proteins) database to infer possible protein-protein interactions, sub-cellular locations, and major altered pathways [1].

Western blot

HCT116 and HCT116 DR cells were washed with PBS, collected, centrifuged at 500× *g* for 5 min and resuspended in lysis buffer [150 mM NaCl, 50 mM Tris (pH 8.0), 5 mM EDTA, 2% (*v/v*) NP-40, 1-phosphatase inhibitor (PhosStop, Roche), 1 tablet of protease inhibitor (cOmplete Mini, Roche), 1 mM PMSF, and 0.1% (*w/v*) DTT]. Whole-cell extracts were sonicated in pulses (10 times, 1 min each) and centrifuged at 8000× *g* for 10 min. The supernatant was recovered, and protein concentration was determined using the Pierce 660nm Protein Assay Reagent (ThermoFisher Scientific) according with manufacturer's specifications. Then, 25 μg total protein extracts were separated by sodium dodecyl sulphate polyacrylamide gel electrophoresis (SDS-PAGE) in a 7.5% (37.5:1) acrylamide-bisacrylamide gel (Merck Millipore). The electrophoretic transfer was performed for 24 h onto a 0.45-mm Polyvinylidene difluoride (PVDF) membrane (GE Healthcare), in Tris-buffered saline with 0.1% (*v/v*), followed by blocking with 5% (*w/v*) milk solution in Tris-buffered saline with 0.1% (*v/v*) Tween 20 (TBST) for 1 h. After, the membrane was incubated for 24 h at 4 °C with primary antibody against EGFR (EGFR (D-8), sc-365829, Santa Cruz), and for 1 h at room temperature against β-actin (reference no. A5441, Sigma). Membranes were washed with TBST and incubated with the appropriate secondary antibody conjugated with horseradish peroxidase (reference no. 7074 or 7076, Cell Signaling Technology). WesternBright ECL (Advansta) was applied to the membranes, and signal was acquired in a Gel Doc imager (Bio-Rad).

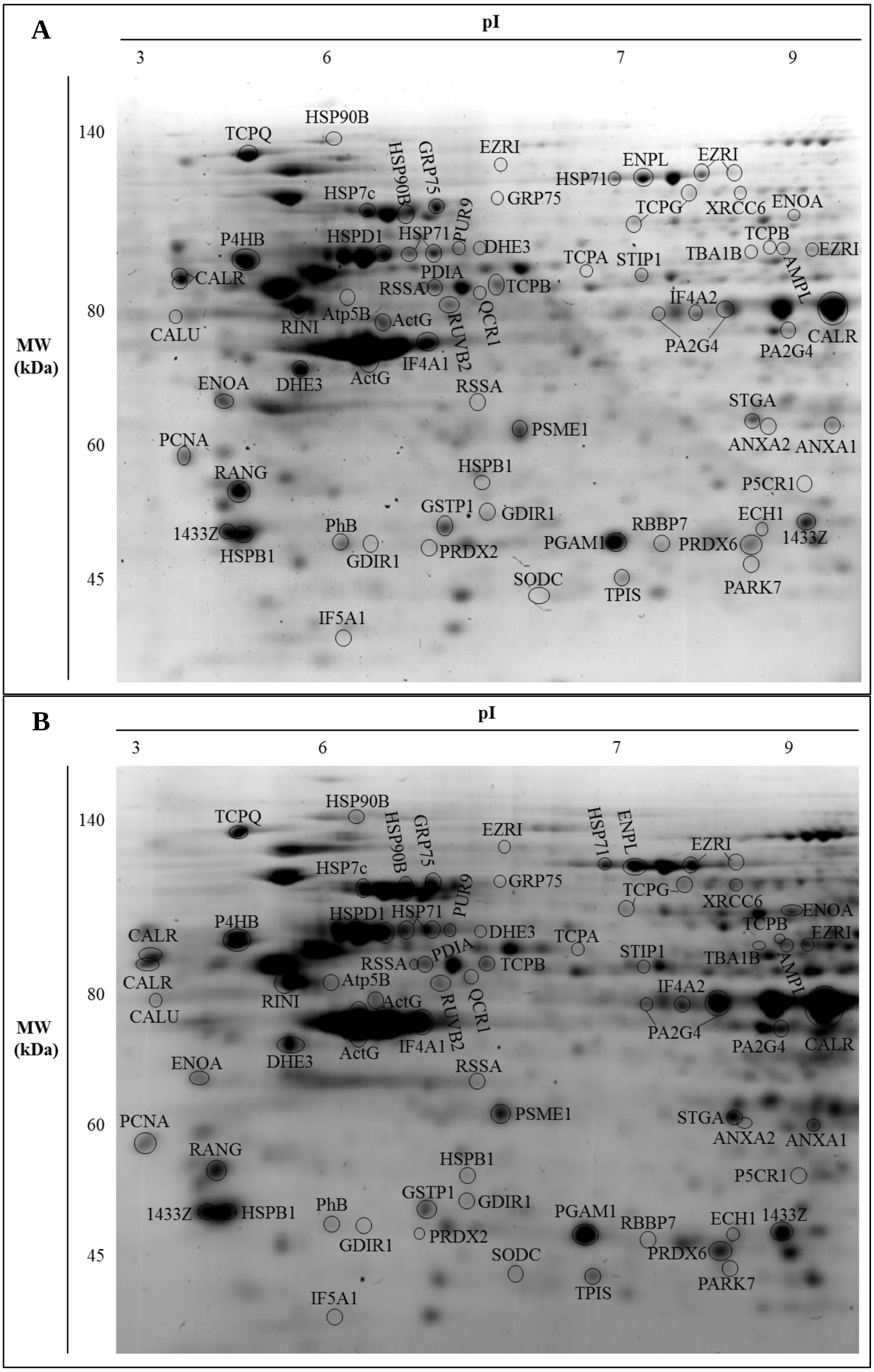

Figure 4. - 2-DE proteome map of: A. HCT116 DR untreated cells and B. HCT116 DR treated during 48 h with ZnD (IC_{50}). Proteins were separated by isoelectric focusing (pI 3-10 non-linear) in the first dimension and SDS-PAGE in the second dimension. Visualization was done by staining with Coomassie brilliant blue R-350. Most relevant identified proteins are mentioned. 2-DE gel images are used in compliance with the digital image and integrity policies (www.nature.com/srep/policies/index.html#digital-image). No grouping has been made. Original full-length gels are shown. No changes in contrast (exposure) were made.

Protein extracts from 48 h-treatment with ZnD and untreated HCT116 DR cells were subjected to proteomic analysis to explore the main molecular players involved in the response to ZnD. The most abundant proteins with isoelectric points ranging from 3 to 10 were the focus of the proteomic analysis performed through a two-dimensional electrophoresis (2-DE). For each condition, more than 500 protein spots were detected (Figure S4) and to distinguish differences in protein expression, the fold variation between ZnD-treated HCT116 DR cells and the corresponding control was calculated (Table S1). Fold variations were considered significant if they were below 0.7 (down-regulated proteins) or above 1.5 (up-regulated proteins) with a p-value bellow 0.05, when compared to control cells. The 33 proteins that were most differentially expressed identified are described (Figure S4 and Table S1).

Table S1. Fold variance of proteins between HCT116 DR cells treated for 48 h with ZnD compared with untreated controls.

Uniprot Kb Identifier	Gene name	Protein name	HCT116 DR+ZnD/HCT116 DR
**P10809**	**HSPD1**	60 kDa heat shock protein, mitochondrial	1.6
P38646	GRP75	Stress-70 protein, mitochondrial	1.5
P0DMV8	HSPA1A	Heat shock 70 kDa protein 1A	1.5
P08238	HSP90B	Heat shock protein HSP 90 – beta	1.7
P14625	GRP94	Endoplasmin	1.7
P30041	PRDX6	Peroxiredoxin-6	1.7
P00441	SOD1	Superoxide dismutase [Cu-Zn]	1.8
P12956	XRCC6	X-ray repair cross-complementing protein 6	2.2
P09211	GSTP1	Glutathione S-transferase P	0.7
P32119	PRDX2	Peroxideroxin-2	0.2
P04792	HSPB1	Heat shock protein beta-1	0.50
P00367	GLUD1	Glutamate dehydrogenase 1, mitochondrial	0.6
P31939	ATIC	Bifunctional purine biosynthesis protein PURH	1.9
Q13011	ECH1	Delta(3,5)-Delta(2,4)-dienoyl-CoA isomerase, mitochondrial	0.6
P06733	ENO1	Alpha-enolase	1.5
P15311	EZR	Ezrin	4.4
P35232	PHB	Prohibitin	0.4
P63241	EIF5A	Eukaryotic translation initiation factor 5A-1	0.3
P63104	YWHAZ	14-3-3 protein zeta/delta	0.7
P12004	PCNA	Proliferating cell nuclear antigen	0.6
P78371	CCT2	T-complex protein 1 subunit beta	0.7
P49368	CCT3	T-complex protein 1 subunit gamma	0.6
P17987	TCP1	T-complex protein 1 subunit alpha	0.6
P43487	RANBP1	Ran-specific GTPase-activating protein	0.6
P31948	STIP1	Stress-induced-phosphoprotein 1	0.6
P52565	ARHGDIA	Rho GDP-dissociation inhibitor 1	0.5
P60174	TPI1	Triosephosphate isomerase	0.6
Q16576	RBBP7	Histone-binding protein RBBP7	0.3
P04083	ANXA1	Annexin A1	1.6
P27797	CALR	Calreticulin	1.8
O43852	CALU	Calumenin	1.8
P28838	LAP3	Cytosol aminopeptidase	2.2
P08865	RPSA	40S ribosomal protein SA	1.9

ZnD-treated cells exhibited overexpression of heat shock proteins (HSPs) namely HSPD1, GPR94, GRP75, HSPA1A, and HSP90B. The HSPs are a heterogeneous group of chaperones which functions include the protection of cells from environmental stress damage through a protein quality control mechanism and the cooperation in newly synthesized polypeptides transport to target organelles [2,3]. The high intracellular levels of GRP94, GRP75, HSPA1A, HSP90B and HSPD1 could indicate a possible accumulation of reactive oxygen species in the cells, culminating in cell death. These results agree with an increase in PRDX6, SOD1 levels. These enzymes are involved in cellular protection against oxidative injury [3], suggesting that HCT116 DR cells tries to cope with the induction of reactive oxygen species (ROS) when exposed to ZnD. Interestingly, PRDX2 and GSTP1 that are usually overexpressed in colorectal carcinoma cells [4,5] and are important for tumorigenesis, are reduced in the presence of ZnD. XRCC6 is involved in non-homologous end-joining and ATIC1 is involved in de novo purine biosynthesis and their up-regulation, in the presence of ZnD, might indicate that HCT116 DR cells are trying to counteract the damage induced by the compound [6,7].

HSPB1 is a "survival protein" that interferes with several cell death pathways, specifically in upstream events (e.g., the release of cytochrome-c) of the apoptotic cascade and its levels are reduced in response to ZnD promoting its antiproliferative action [8].

GLUD1 has a pivotal role in nutritional stress and is associated with tumor aggressiveness and poorer prognosis in colorectal cancer and is an important target in the treatment of refractory colorectal cancer [9]. Interestingly, GLUD1 level are decreased in HCT116 DR cells after exposure to ZnD indicating the capability of the compound to cope with tumor aggressiveness.

Reduced EZR expression plays a critical role in the development, epithelial-mesenchymal transition induction and metastasis formation of colorectal carcinoma tumors [10]. ENO1 is a bifunctional protein acting as a glycolytic enzyme and a transcription factor. By repressing c-Myc gene and in case of acting as a transcription factor, ENO1 plays a critical role in cancer inhibition [11]. PHB overexpression has been also correlated with cancer. Considering this, the exposure of HCT116 DR cells to ZnD allows to counteract these effects via EZR and ENO1 up-regulation and PHB down-regulation.

EIF5A has been associated with cell proliferation and it can serve as a marker for malignant growth [12]. 1433Z is involved in the regulation of several signaling pathways including cell proliferation, cell apoptosis and angiogenesis. The overexpression of 1433Z can be positively correlated with cancer progression, metastasis and worse survival in patients [13]. PCNA is at the very heart of many essential cellular processes, such as DNA replication, repair of DNA damage, chromatin structure maintenance, chromosome segregation and cell-cycle progression [14]. Interestingly, all these proteins are repressed in the presence of ZnD which could be correlated with its antiproliferative/apoptotic effect (Figure 2).

ANXA1 belongs to the family of phospholipid and calcium binding proteins, being involved in the anti-inflammatory process, regulation of differentiation, proliferation and apoptosis [15]. It has been described that increased expression of this protein promotes apoptosis with the activation of caspase-3 [16,17]. CALU and CALR are Ca^{2+} binding proteins that are normally found in the endoplasmic reticulum. However, CALU can be transported into the cytoplasm after cell cycle arrest or in late apoptosis and CALR can be translated to cell surface and functioning as an “eat-me” signal during end stages of apoptosis [18–20]. ANXA1, CALR and CALU are overexpressed in the presence of ZnD, which may indicate its ability to induce the apoptotic process as previously observed and also indicating that HCT116 DR cells are mostly found in late apoptosis (Figure 2) [18,19].

CCT2, CCT3, TCP1 are cytoskeletal proteins, including tubulins, actin, and proteins involved in the control of cell cycle [21]. Thus, their down-regulation in the presence of ZnD might be correlated with a delay in the cell cycle progression and confirming the cytostatic potential of this compound (see also Figure 3 in main manuscript).

5. Expression of EGFR in tumor and normal cells lines

A

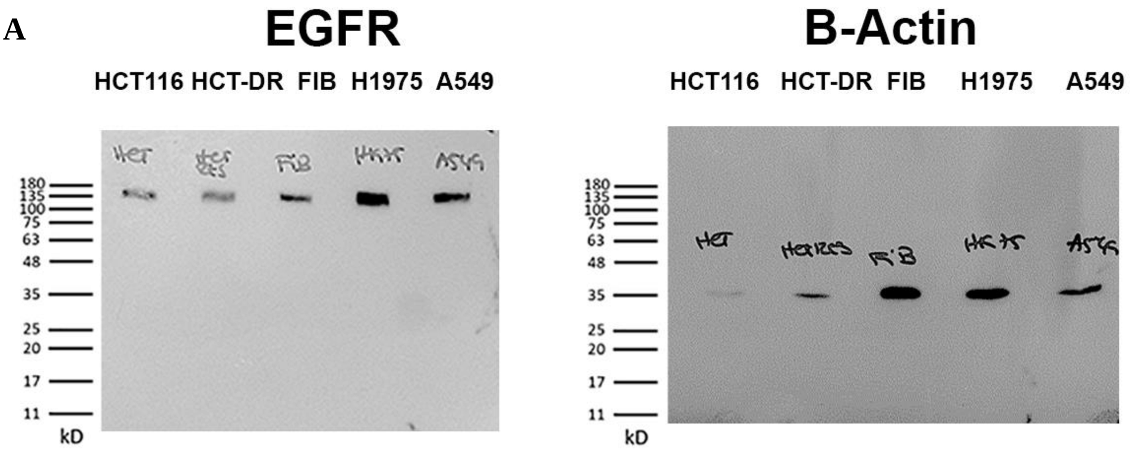

B

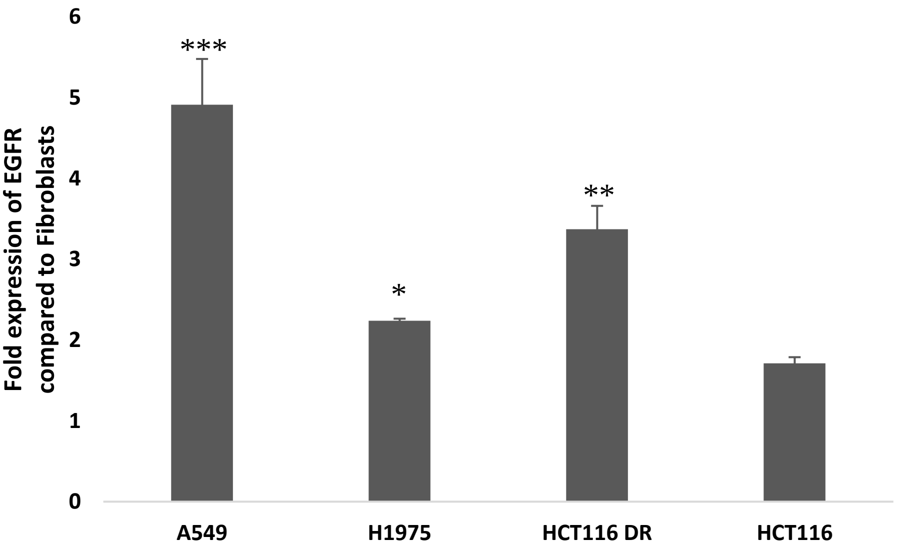

Figure S5. Expression of EGFR in A549, H1975, HCT116 DR and HCT116 cells compared to Fibroblasts. A. EGFR (172 kDa) and β-actin (42 kDa) protein quantification was performed via western blotting on cell extracts. Western blots images are used in compliance with the digital image and integrity policies (www.nature.com/srep/policies/index.html#digital-image). No grouping of western blots has been made. Original full-length blots are shown. No changes in contrast (exposure) were made. Please disregard the handwritten notes on the radiographies. B. Fold expression of EGFR was compared with fibroblast expression for reach cell line. Band quantification was normalized against ACTB. The results are expressed as the mean ± SD percentage normalized to controls from two independent experiments (* $P < 0.05$; ** $P < 0.01$; *** $P < 0.001$).

6. Synthesis and Characterization of AuNPs conjugates and ZnD interaction with BSA

Naked AuNPs were synthetized according to Meisel et al [22]. with an average diameter of 14 nm that were characterized by TEM, UV-Spectroscopy and DLS (Figure 4 and Table 2). These naked AuNPs were subsequently functionalized with an excess HS-PEG-COOH to attain 100% surface coverage (AuNP@PEG) and thus, promote nanoconjugate stabilization and biocompatibility. AuNP@PEG were further coupled to bovine serum albumin (BSA) and Cetuximab (anti-EGFR antibody) via carbodiimide chemistry (EDC/NHS coupling reaction) [23]. After each functionalization step, nanoconjugates were characterized by UV/Vis spectroscopy and DLS, both indicating an increase to the radii of NPs and a change to the surface's dielectric, consistent with functionalization at the NPs' surface (Figure 4C and D and Table 2). Bradford assay revealed a functionalization efficiency of approximately 7 BSAs per gold nanoparticle (BSA:AuNPs ≈ 7). BSA is bound to the AuNP via covalent binding between amine groups present in both proteins and the PEG carboxyl group. AuNP@PEG were also functionalized with Cetuximab (AuNP@PEG@CETUX) and quantification of the supernatants using the Bradford assay reveal 2 Cetuximab per AuNP (NCetuximab:NAuNPs ≈2). This AuNP@PEG@CETUX were subsequently functionalized with BSA AuNP@PEG@CETUX@BSA; (BSA:AuNPs ≈ 7).

Serum albumin is the most abundant protein in blood plasma. This protein provides the intracellular binding, transportation and delivery of endogenous and exogenous compounds, such as fatty acids, steroids, metal compounds, metabolites and several pharmaceutical agents [24–26]. It is well known that the ability of serum albumin to bind non-covalently with small molecules, results in an increased solubility of ligands in plasma and enhanced delivery to the target site of these molecules [24–26]. Taking advantage of the intrinsic fluorescence of BSA due to its tryptophan residues, the fluorescence quenching of these proteins at various concentrations of ZnD was studied (Figure S6).

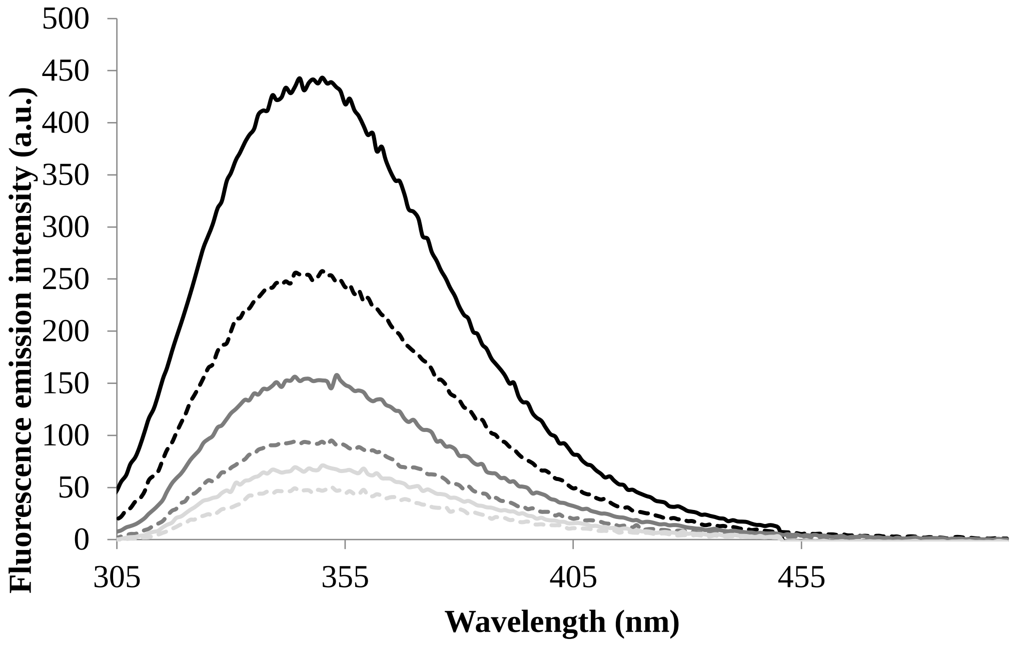

Figure S6. Fluorescence emission spectra of BSA (0.13 µM; black line) excited at 280 nm with different concentrations of ZnD (0 µM – black line, 75 µM - black dashed line, 175 µM– dark grey line, 250 µM– dark grey dashed line, 300 µM– light grey line, 400 µM – light grey dashed line), incubated for 1 hour at 37 °C. Data is representative of at least three different assays.

In the absence of ZnD, the maximum emission wavelength observed at 350 nm (black line in Supplementary Figure S5) can be attributed only to the intrinsic fluorescence of BSA molecule. The effect of ZnD on spectroscopic properties of the BSA molecule was very pronounced, in which there was a remarkable decrease of BSA intrinsic fluorescence with the increase of compound concentration, reaching a decrease of about 89% at the highest ZnD concentration used (i.e. 400 µM). These results confirmed that the compound act as a quencher of the intrinsic fluorescence of BSA and an interaction between ZnD and BSA exists.

It has been described that the ability of albumin to associate non-covalently with smaller molecules and ions is due to the existence of at least six binding regions in the protein, two high affinity binding sites, situated in Sudlow's sites I and II as previously described, and other sites with lower affinity for ligands [27]. The fact that there are only two high affinity sites for the binding of ligands to BSA (situated in Sudlow's sites I and II), suggests that almost all ZnD molecules are bound to the large number of weaker binding sites. The interactions of ZnD with these lower affinity sites for ligands could be also supported by the fact of the quenching of the tryptophan residues fluorescence was very strong at lower molar ratios, but in higher molar ratios it progressed weakly, suggesting weaker binding sites for ZnD, and probably located away from the tryptophan residues. The nature of the interaction between ZnD and BSA could be electrostatic since the isoelectric point of BSA is 4.7 [28] and in the experimental pH (~7), BSA possesses a negative charge whereas the compounds are positive charged. In these fluorescence studies, BSA was in its native conformation. Proving this interaction between ZnD and BSA, AuNP@PEG@BSA and AuNP@PEG@CETUX@BSA were functionalized with ZnD and the amount of ZnD remaining in the supernatants quantified via UV-spectroscopy and ICP-MS (to calculate the number of ZnD molecules loaded into the nanoformulations – Table 2). The AuNPs@PEG@BSA (7 BSA molecules per AuNP) were incubated with 50 µM of ZnD, and a maximum of 57 ZnD molecules bound to the protein – ICP-AES data show that 402 ± 32 molecules of ZnD per AuNP. It is believed that the BSA molecules covalently linked to PEG on the AuNPs surface using the EDC/sulfo NHS reaction suffer some degree of denaturation. However, these biding assays using fluorescence, refer to BSA in its native conformation, which allow for higher molar ratios of ZnD:BSA, indicating that each BSA molecule could interact with a higher number of ZnD molecules. In fact, binding of BSA to PEG in AuNPs@PEG@BSA may trigger an

altered tertiary structure, leading to steric hindrance of several binding sites, which in turn would result in less molecules interacting with BSA than in its free status (native conformation). No interaction of ZnD with naked AuNP or AuNP@PEG was observed after a 1h incubation (results not shown). Upon loading of ZnD onto AuNPs@PEG@CETUXI@BSA, a lower number of BSA molecules were bound to the surface to give room for cetuximab (1.6 molecules per AuNP). ICP-AES data show a ratio of 432 ZnD molecules per AuNP, meaning that we cannot discard that some ZnD might also be interacting with cetuximab.

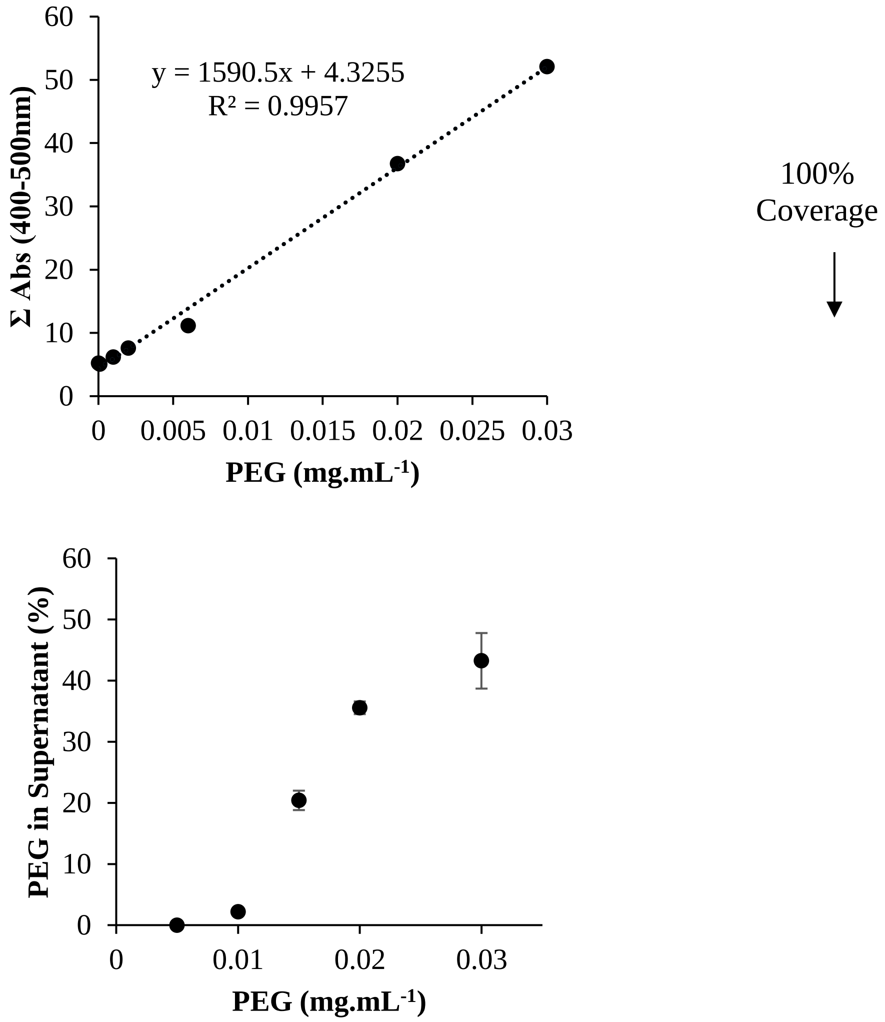

Figure S7. Characterization of PEG functionalization at AuNPs surface. (Left) Calibration curve of Elman's assay for amount of PEG and absorption at 400–500 nm. (Right) Percentage of PEG measured in the supernatant compared with PEG added to the AuNPs.

7. Internalization of AuNP@PEG@CETUX in HCT116

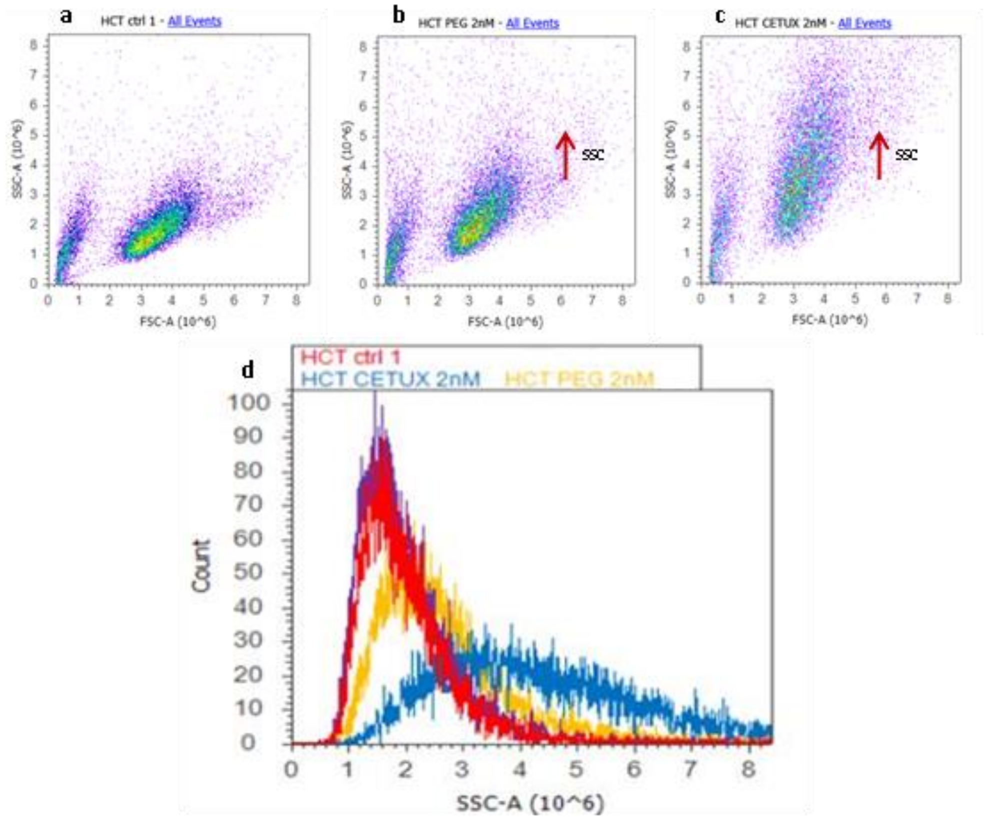

Figure S8. Forward and side scatter profiles at 488 nm of HCT116 cells after being exposed during 6 h to fresh growth medium (**a**), and to the nanoparticle formulations, AuNP@PEG (**b**) and AuNP@PEG@CETUX (**c**). Histogram plot evidencing the scattering profiles of HCT-116 cells when exposed to nanoparticle constructs (**d**). FSC-A - Forward Scatter; SSC-A-Side Scatter.

8. Cytotoxicity of nanoconjugates in EGFR overexpressing cell lines

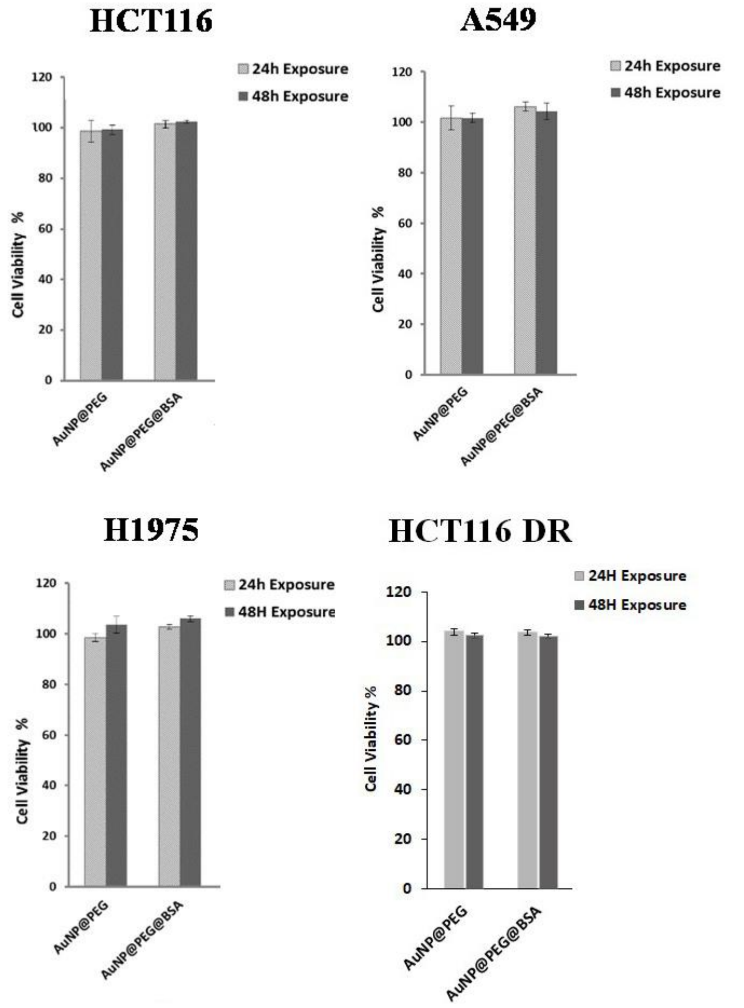

Figure S9. Cell viability assessment of the control nanoconjugates in HCT116, HCT116 DR, A549, H1975 cell lines after 24 and 48 h incubation. Concentration of control nanoconjugates used correspond to same concentration of Au as in nanoparticles functionalized with ZnD (IC_{50} at 48 h). Data represented as mean ± SEM of at least three independent experiments. Cell viability was normalized to the control cells without nanoparticles.

Among the tested cell lines, the ZnD nanoconjugates (using IC_{50} for ZnD) induces a higher cytotoxicity in lung adenocarcinoma cell lines (A549 and H1975) than colorectal (HCT116 and HCT116 DR) - Figure 5; Figure S8 and Table S2. Also, lung adenocarcinoma cells with L858R/T790M-EGFR mutations (H1975) are more susceptible to ZnD than Wild Type EGFR (A549) (IC_{50} = 0.355 ± 0.04 μM and 0.714 ± 0.09 μM, respectively) - Table S2.

Table S2. Cell viability (%) obtained for HCT116, HCT11 DR, A549 and H1975 cell lines for free ZnD and the nanoconjugates' formulations evaluated 24 and 48 h after exposure with the correspondent IC_{50} at 48 h for each cell line (Table 1). Data are expressed as mean ±SEM of at least three independent experiments.

**Nanoparticle/Drug Formulation**		**Cell viability (%) (± SEM)**	
		**24 h**	**48 h**
**HCT116**	AuNP@PEG@BSA@ZnD	43.8 (± 1.4)	31.3 (± 1.0)
	NanoZnD	42.3 (± 2.1)	34.5 (± 1.7)
	ZnD free	61.2 (± 1.3)	56.0 (± 1.5)
HCT116 DR	AuNP@PEG@BSA@ZnD	49.4 (± 1.3)	37.7 (± 1.3)
	NanoZnD	43.1 (± 1.3)	41.1 (± 2.0)
	ZnD free	64.0 (± 1.3)	55.6 (± 2.0)
A549	AuNP@PEG@BSA@ZnD	20.2 (± 6.9)	13.1 (± 2.5)
	NanoZnD	21.8 (± 4.5)	12.2 (± 0.3)
	ZnD free	64.3 (± 7.4)	44.9 (± 3.3)
H1975	AuNP@PEG@BSA@ZnD	30.3 (± 4.1)	20.4 (± 2.9)
	NanoZnD	31.3 (± 3.5)	22.5 (± 5.1)
	ZnD free	87.4 (± 7.0)	67.8 (± 12.6)

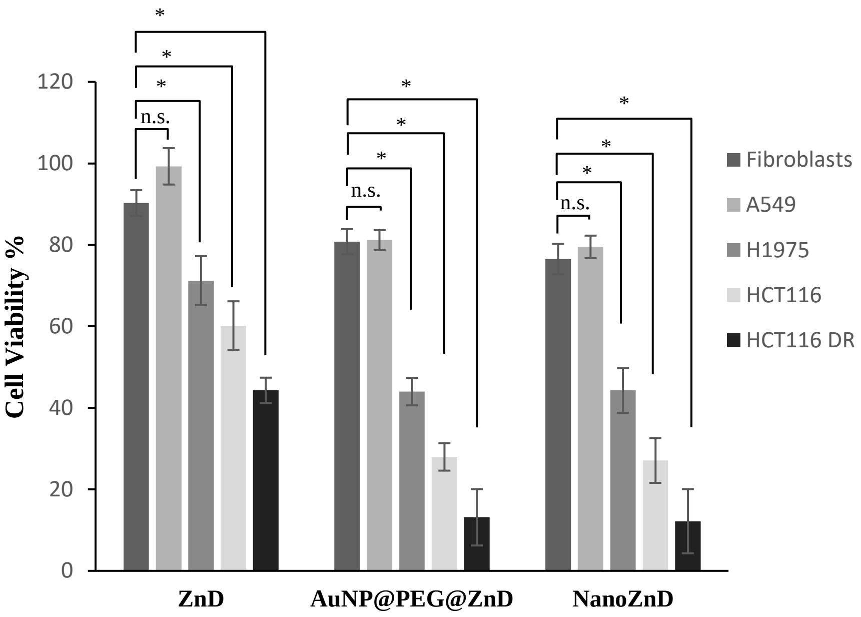

Figure S10. Cell viability assessment of Free ZnD and nanoconjugates in fibroblasts, A549, H1975, HCT116 and HCT116 DR cell lines after 48 h incubation. Concentration of ZnD nanoconjugates correspond to same concentration of free ZnD (0.355 μM). Data represented as mean ± SEM of at least three independent experiments. Cell viability was normalized to the control cells without nanoparticles.

References

1. Gomes, S.E.; Pereira, D.M.; Roma-Rodrigues, C.; Fernandes, A.R.; Borralho, P.M.; Rodrigues, C.M.P. Convergence of miR-143 overexpression, oxidative stress and cell death in HCT116 human colon cancer cells. *PLoS ONE* **2018**, *13*, e0191607.
2. Sarto, C.; Binz, P.-A.; Mocarelli, P. Heat shock proteins in human cancer. *Electrophoresis* **2000**, *21*, 1218–1226.
3. Karsani, S.A.; Saihen, N.A.; Zain, R.B.; Cheong, S.-C.; Rahman, M.A. Comparative proteomics analysis of oral cancer cell lines: Identification of cancer associated proteins. *Proteome Sci.* **2014**, *12*, 3.
4. Nicolussi, A.; D'Inzeo, S.; Capalbo, C.; Giannini, G.; Coppa, A. The role of peroxiredoxins in cancer. *Mol. Clin. Oncol.* **2017**, *6*, 139–153.
5. Dang, D.T.; Chen, F.; Kohli, M.; Rago, C.; Cummins, J.M.; Dang, L.H. Glutathione S-Transferase π1 Promotes Tumorigenicity in HCT116 Human Colon Cancer Cells. *Cancer Res.* **2005**, *65*, 9485–9494.
6. Fell, V.L.; Schild-Poulter, C. The Ku heterodimer: Function in DNA repair and beyond. *Mutat. Res. Rev. Mutat. Res.* **2015**, *763*, 15–29.
7. Yin, J.; Ren, W.; Huang, X.; Deng, J.; Li, T.; Yin, Y. Potential Mechanisms Connecting Purine Metabolism and Cancer Therapy. *Front. Immunol.* **2018**, *9*, 1697.
8. Gibert, B.; Hadchity, E.; Czekalla, A.; Aloy, M.-T.; Colas, P.; Rodriguez-Lafrasse, C.; Arrigo, A.-P.; Diaz-Latoud, C. Inhibition of heat shock protein 27 (HspB1) tumorigenic functions by peptide aptamers. *Oncogene* **2011**, *30*, 3672–3681.
9. Miyo, M.; Konno, M.; Nishida, N.; Sueda, T.; Noguchi, K.; Matsui, H.; Colvin, H.; Kawamoto, K.; Koseki, J.; Haraguchi, N.; et al. Metabolic Adaptation to Nutritional Stress in Human Colorectal Cancer. *Sci. Rep.* **2016**, *6*, 38415.
10. Haynes, J.; Strivastava, J.; Madson, N.; Wittmann, T.; Barber, D.L. Dynamic actin remodeling during epithelial–mesenchymal transition depends on increased moesin expression. *Mol. Biol. Cell* **2011**, *22*, 4750–4764.
11. Milone, M.R.; Pucci, B.; Colangelo, T.; Lombardi, R.; Iannelli, F.; Colantuoni, V.; Sabatino, L.; Budillon, A. Proteomic characterization of peroxisome proliferator-activated receptor-γ (PPARγ) overexpressing or silenced colorectal cancer cells unveils a novel protein network associated with an aggressive phenotype. *Mol. Oncol.* **2016**, **10**, 1344–1362.
12. Mathews, M.B.; Hershey, J.W.B. The translation factor eIF5A and human cancer. *Biochim. Biophys. Acta* **2015**, *1849*, 836–844.

13. Hodgkinson, V.C.; ELFadl, D.; Agarwal, V.; Garimella, V.; Russell, C.; Long, F.D.; Fox, J.N.; McManus, P.L.; Mahapatra, T.K.; Kneeshaw, P.J.; et al. Proteomic identification of predictive biomarkers of resistance to neoadjuvant chemotherapy in luminal breast cancer: A possible role for 14-3-3 theta/tau and tBID? *J. Proteomics* **2012**, *75*, 1276–1283.
14. Stoimenov, I.; Helleday, T. PCNA on the crossroad of cancer. *Biochem. Soc. Trans.* **2009**, *37*, 605–613.
15. Lecona, E.; Barrasa, J.I.; Olmo, N.; Llorente, B.; Turnay, J.; Lizarbe, M.A. Upregulation of Annexin A1 Expression by Butyrate in Human Colon Adenocarcinoma Cells: Role of p53, NF-Y, and p38 Mitogen-Activated Protein Kinase. *Mol. Cell. Biol.* **2008**, *28*, 4665–4674.
16. Parente, L.; Solito, E. Annexin 1: More than an anti-phospholipase protein. *Inflamm. Res.* **2004**, *53*, 125–132.
17. Beck, H.C.; Petersen, J.; Nielsen, S.J.; Morszeck, C.; Jensen, P.B.; Sehested, M.; Granslund, M. Proteomic profiling of human colon cancer cells treated with the histone deacetylase inhibitor belinostat. *Electrophoresis* **2010**, *31*, 2714–2721.
18. Bull, V.H.; Fargestad, E.M.; Strozynski, M.; Thiede, B. Temporal proteome profiling of taxol-induced mitotic arrest and apoptosis. *Electrophoresis* **2010**, *31*, 1873–1885.
19. Huang, Y.-H.; Chang, A.Y.W.; Huang, C.-M.; Huang, S.-W.; Chan, S.H.H. Proteomic analysis of lipopolysaccharide-induced apoptosis in PC12 cells. *Proteomics* **2002**, *2*, 1220–1228.
20. Lu, Y.-C.; Weng, W.-C.; Lee, H. Functional Roles of Calreticulin in Cancer Biology. *BioMed Res. Int.* **2015**, *2015*, 526524.
21. Coghlin, C.; Carpenter, B.; Dundas, S.R.; Lawrie, L.C.; Telfer, C.; Murray, G.I. Characterization and over-expression of chaperonin t-complex proteins in colorectal cancer. *J. Pathol.* **2006**, *210*, 351–357.
22. Lee, P.C.; Meisel, D. Adsorption and surface-enhanced Raman of dyes on silver and gold sols. *J. Phys. Chem.* **1982**, *86*, 3391–3395.
23. Conde, J.; Ambrosone, A.; Sanz, V.; Hernandez, Y.; Marchesano, V.; Tian, F.; Child, H.; Berry, C.C.; Ibarra,M.R.; Baptista, P.V.; et al. Design of multifunctional gold nanoparticles for in vitro and in vivo gene silencing. *ACS Nano* **2012**, *6*, 8316–8324.
24. Larsen, M.T.; Kuhlmann, M.; Hvam, M.L.; Howard, K.A. Albumin-based drug delivery: Harnessing nature to cure disease. *Mol. Cell. Ther.* **2016**, *4*, 3.
25. Trnkova, L., Bousova, I., Kubicek, V.; Drsata, J. Binding of naturally occurring hydroxycinnamic acids to bovine serum albumin. *Nat. Sci.* **2010**, *2*, 563–570.
26. Sankareswari, V.G.; Vinod, D.; Mahalakshmi, A.; Alamelu, M.; Kumaresan, G.; Ramaraj, R.; Rajagopal, S. Interaction of oxovanadium(iv)–salphen complexes with bovine serum albumin and their cytotoxicity against cancer. *Dalt. Trans.* **2014**, *43*, 3260–3272.
27. Shahabadi, N.; Maghsudi, M.; Ahmadipour, Z. Study on the interaction of silver(I) complex with bovine serum albumin by spectroscopic techniques. *Spectrochim. Acta Part A Mol. Biomol. Spectrosc.* **2012**, *92*, 184–188.
28. Chaudhary, A.; Gupta, A.; Khan, S.; Nandi, C.K. Morphological effect of gold nanoparticles on the adsorption of bovine serum albumin. *Phys. Chem. Chem. Phys.* **2014**, *16*, 20471–20482.
